# Cortico-striatal network architecture shapes rhythmic biases in music

**DOI:** 10.1093/cercor/bhag091

**Published:** 2026-10-06

**Authors:** Massimo Lumaca, Jelle van der Werff, Peter E Keller, Sonja A Kotz, Giosuè Baggio, Mattia Rosso, Marcelo Kakihara, Mathilde Martin, Peter Vuust, Andrea Ravignani

**Affiliations:** Center for Music in the Brain, Aarhus University & The Royal Academy of Music Aarhus/Aalborg, Universitetsbyen 3, Building 1710, 8000 Aarhus C, Denmark; Department of Human Neurosciences, Sapienza University of Rome, Viale dell'Università 30, 00185 Rome, Italy; Center for Music in the Brain, Aarhus University & The Royal Academy of Music Aarhus/Aalborg, Universitetsbyen 3, Building 1710, 8000 Aarhus C, Denmark; The MARCS Institute for Brain, Behaviour and Development, Western Sydney University, Locked Bag 1797, Penrith NSW 2751, Australia; Faculty of Psychology and Neuroscience, Department of Neuropsychology and Psychopharmacology, Maastricht University, Universiteitssingel 40, 6229 ER Maastricht, The Netherlands; Department of Neurology, Max Planck Institute for Human Cognitive and Brain Sciences, Stephanstraße 1a, 04103 Leipzig, Germany; Language Acquisition and Language Processing Lab, Norwegian University of Science and Technology, Edvard Bulls veg 1, 7491 Trondheim, Norway; Center for Music in the Brain, Aarhus University & The Royal Academy of Music Aarhus/Aalborg, Universitetsbyen 3, Building 1710, 8000 Aarhus C, Denmark; PEM Institute for Systematic Musicology, Ghent University, Miriam Makebaplein 1, 9000 Ghent, Belgium; Kyoto University, Graduate School of Advanced Integrated Studies in Human Survivability, Yoshida-Nakaadachicho 1, Sakyo-ku, Kyoto 606-8306, Japan; ESPCI Paris, PSL Research University, 10 rue Vauquelin, 75005 Paris, France; Center for Music in the Brain, Aarhus University & The Royal Academy of Music Aarhus/Aalborg, Universitetsbyen 3, Building 1710, 8000 Aarhus C, Denmark; Center for Music in the Brain, Aarhus University & The Royal Academy of Music Aarhus/Aalborg, Universitetsbyen 3, Building 1710, 8000 Aarhus C, Denmark; Department of Human Neurosciences, Sapienza University of Rome, Viale dell'Università 30, 00185 Rome, Italy; Research Center of Neuroscience "CRiN-Daniel Bovet", Sapienza University of Rome, Piazzale Aldo Moro 5, 00185 Rome, Italy; Institute of Cognitive Sciences and Technologies, National Research Council, Via Giandomenico Romagnosi 18a, 00196, Rome, Italy; Comparative Bioacoustics Group, Max Planck Institute for Psycholinguistics, Wundtlaan 1, 6525 XD Nijmegen, The Netherlands

**Keywords:** cortico-striatal circuits, functional connectivity, rhythmic biases, white matter microstructure

## Abstract

Why is human music rhythmically structured the way it is? One hypothesis is that the human brain is intrinsically wired to favor specific rhythmic organizations. To test this, we collected diffusion and resting-state functional MRI (fMRI) data from musically untrained participants and, weeks later, asked them to imitate randomly generated drumming sequences using finger taps. These imitations exhibited systematic biases toward temporal structures widespread in human music: predictable patterns characterized by isochronous organization (1:1) and other small integer ratios (2:1 and 3:1). Functional connectome analyses linked the increased rhythmic predictability of reproduced rhythms to intrinsic interactions between networks implicated in auditory timing (auditory basal ganglia) and executive control (task-positive and task-negative networks). Notably, the tendency toward isochrony—a feature highly conserved across species—was selectively associated with increased fiber density within a cortico-basal ganglia-thalamo-cortical network previously implicated in the perception and production of a metronomic pulse from music (the “beat”). Our findings reveal how stable neural architecture predisposes humans to produce rhythmically structured patterns. This evidence links intrinsic brain circuitry to individual cultural expressions, supporting the account of human symbolic systems, including music, as biocultural hybrids.

## Introduction

Humans exhibit tendencies (“biases”) to systematically distort temporal information toward more regular forms when perceiving, imitating, or reproducing musical rhythms. When tasked to replicate randomly timed drumming sequences, even nonmusicians, regardless of cultural background and age (Drake and Bertrand 2001), spontaneously transform the original input into more structured and predictable patterns (Povel 1981; Repp et al. 2012a). These regularization biases vary in detectability across experimental paradigms. In single-reproduction designs, where participants reproduce random rhythms without iteration, individual biases remain subtle and exhibit substantial between-participant variability (Repp et al. 2012b). By contrast, iterated reproduction (Jacoby and McDermott 2017; Jacoby et al. 2024) (participants reproduce their own previous output across multiple iterations) and serial reproduction (outputs are transmitted to the next participant in a transmission chain) (Ravignani et al. 2016; Lumaca and Baggio 2018; Lumaca et al. 2018a; Verhoef and Ravignani 2021) amplify weak individual biases into robust population-level patterns through cumulative cultural filtering. The resulting patterns in iterated and serial reproduction mirror those observed across musical traditions worldwide: predictable sequences characterized by low entropy, featuring isochronous organization (1:1) and other small-integer interval ratios (2:1 and 3:1) (Savage et al. 2015). However, the neurobiological mechanisms underlying these perceptuo-motor biases and their individual variability remain poorly understood (Fitch 2017).

Biases in rhythm perception and production are commonly explained in terms of mode-locking within dynamical-systems frameworks: the tendency of coupled nonlinear systems to settle into stable phase relationships at small integer frequency ratios. Critically, canonical models of neural oscillations make only general assumptions about underlying neural structures. As a result, their predictions apply to an entire family of dynamical systems rather than to any specific neural configuration (Large et al. 2015). In the brain, this principle predicts that interactions between auditory and motor populations will steer rhythm perception and production toward isochronous timing and small integer-ratio intervals (≤ 3; ie 1:1, 1:2, 2:1, 1:3, 3:1) (Large and Snyder 2009; Large et al. 2023; Barbero et al. 2025). Current neurocomputational models embed these dynamics within an anatomical framework comprising cortical regions (auditory cortex, premotor cortex, and supplementary motor areas) and subcortical structures (cerebellum and basal ganglia). Together, these areas orchestrate temporal processing and motor coordination (Grahn and Brett 2007; Cannon and Patel 2021). Empirical evidence further suggests that brain activity is organized in oscillatory systems with dominant frequency modes (Keitel and Gross 2016) and frequency-specific networks that dynamically reorganize endogenous oscillations during rhythmic auditory processing (Rosso et al. 2025). Slow oscillations originating in motor networks can orient and constrain auditory perception through active-sensing mechanisms (Morillon et al. 2014; Morillon and Baillet 2017), while phase interactions between endogenous oscillatory systems and external rhythmic input tend to converge toward stable attractor states (Rosso et al. 2023; Baliviera et al. 2025). These attractors make some timing patterns more stable in perception and attention, more readily manifested in performance and synchronization, and easier to acquire across development.

Neuroimaging meta-analyses demonstrate remarkable consistency in the engagement of auditory-motor circuits during rhythm perception, memorization, and reproduction across participants from different cultural backgrounds (Kasdan et al. 2022). This evidence positions auditory-motor circuits as a biological substrate that may account for similar regularization biases observed across cultural contexts, providing a mechanistic explanation for the widespread occurrence of regular temporal patterns among the musical traditions of the world (Trehub 2000). Nevertheless, direct empirical evidence linking processing predispositions for rhythmic structure with the functional and structural organization of the human brain remains lacking. If this hypothesis is correct, individual differences in neural organization (Van Horn et al. 2008; Kanai and Rees 2011) should partly account for differences in these general biases observed across individuals in the general population (Mansuri et al. 2022; Dalla Bella et al. 2024).

In this study, we tested this hypothesis by combining multi-modal neuroimaging with a single-reproduction rhythm task. Forty-seven healthy adults, nonmusicians (Supplementary Fig. S1 and Supplementary Table S1), participated in a two-part experiment involving brain scans and a behavioral finger-tapping task, conducted on separate days (Fig. 1A). After initial MRI acquisition (including resting-state functional, anatomical, and diffusion images), participants returned 6 to 50 d later to reproduce randomly generated drum sequences through finger tapping in a computer-based task. By acquiring neuroimaging data days before behavioral testing, we aimed at isolating the contribution of stable neural architecture to individual rhythmic biases, independent of task-specific plasticity or learning effects. Test–retest neuroimaging studies support the temporal stability of diffusion-based white-matter metrics (Heiervang et al. 2006; Boekel et al. 2017). Resting-state functional connectivity, moreover, shows moderate-to-high reliability (Teeuw et al. 2021), with whole-brain connectivity patterns sufficiently distinctive to identify individuals across sessions with ~90% accuracy (Finn et al. 2015). The temporal gap between MRI and behavioral data acquisition allowed brain structure to serve as a prospective neural predictor, rather than a mere correlate of subsequent behavior (Gabrieli et al. 2015). Without this separation, scanning immediately before the task would risk conflating stable trait-level neural architecture with transient state-level effects such as anticipatory arousal, while scanning immediately after would capture short-term, task-induced modifications of resting-state connectivity (Tung et al. 2013; Gordon et al. 2014) rather than preexisting architecture.

**Figure 1:**
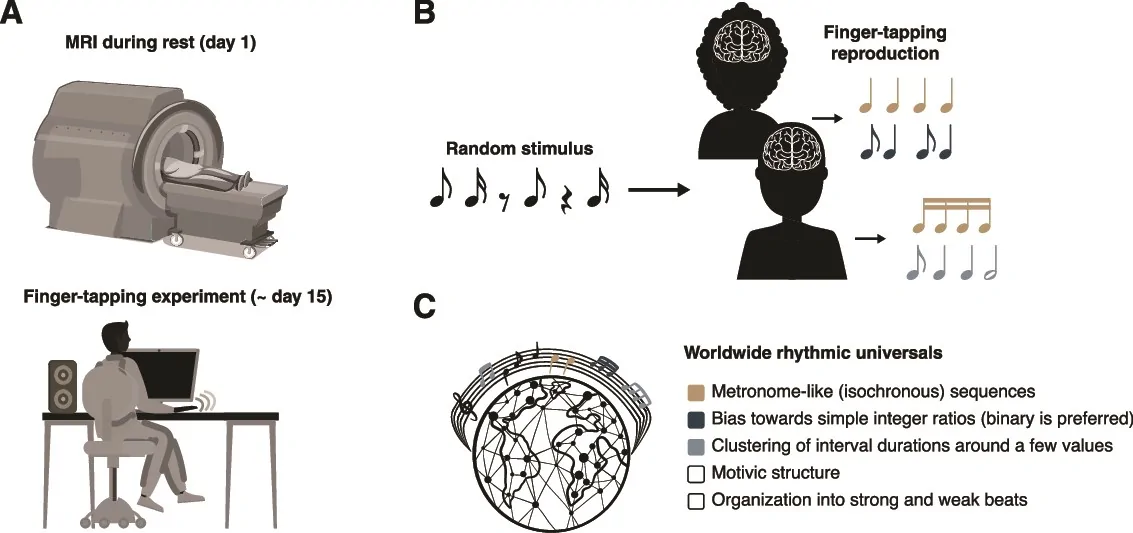
Experimental design and behavioral mapping to rhythmic universals. A) Experimental timeline. Participants underwent resting-state functional and diffusion MRI scanning, followed weeks after (mean: 17.5 d) by a behavioral task in which participants listened to randomly timed sound sequences and subsequently reproduced them via finger tapping. B) Systematic biases in reproduction. Participants showed inter-individual variability and systematic biases toward specific rhythmic features. These biases are color-coded to match the corresponding universals in panel C: introduction of isochronous intervals (tan), introduction of binary and ternary interval ratios (deep blue), and reduction of Shannon entropy (blue gray). C) Mapping of behavioral features to cross-cultural rhythmic universals. The observed biases map to three out of five independently attested rhythmic universals: metronomic patterns, simple integer ratios (2:1 and 3:1), and clustering of interval durations around a few values. Two universals (motific structure and organization into strong and weak beats) were not addressed here due to limitations of our tapping paradigm. The globe icon represents the cross-cultural scope of these rhythmic universals, which have been documented across diverse musical traditions worldwide (Savage et al. 2015).

Our single-reproduction design models a single-generation step in serial reproduction paradigms (Ravignani et al. 2016; Lumaca et al. 2018a) (Fig. 1B). While multi-generation paradigms demonstrate how biases shape cultural evolution, single-reproduction designs offer the advantage to capture individual biases in their raw form, before transmission dynamics obscure person-level heterogeneity. We specifically preferred this approach over within-individual iterated reproduction because iterative refinement may partly conflate intrinsic biases with learning and adaptation effects, introducing variance attributable to short-term plasticity rather than to the stable neural architecture measured days earlier at rest. Single reproduction thus isolates individual perceptuo-motor biases that, when iterated across multiple individuals, lead to the population-level patterns observed in laboratory mini-cultures. We quantified properties of the rhythmic stimuli and finger-tap responses using mathematical measures of rhythmic structure and information content (Shannon 1948; Roeske et al. 2020; Laffi et al. 2026) that capture various features of musical rhythms: isochronous ratios (1:1) and other small-integer interval ratios (1:2, 2:1, 1:3, or 3:1), and the probability distribution of interval durations (entropy) (Fig. 2). Each measure maps onto one of three statistical universals identified across the world’s musical traditions (Savage et al. 2015) (Fig. 1C): isochronous interval ratios capture the prevalence of metronomic patterns; binary and ternary ratios capture the bias toward small integer ratios; and Shannon entropy captures the clustering of interval durations around a few categorical values. Additionally, we used edit distance (Levenshtein 1966) between stimuli and finger-tap responses as a measure of transmission error. These behavioral measures have been previously used in our serial reproduction studies of rhythm evolution (Ravignani et al. 2016; Lumaca et al. 2018a).

**Figure 2:**
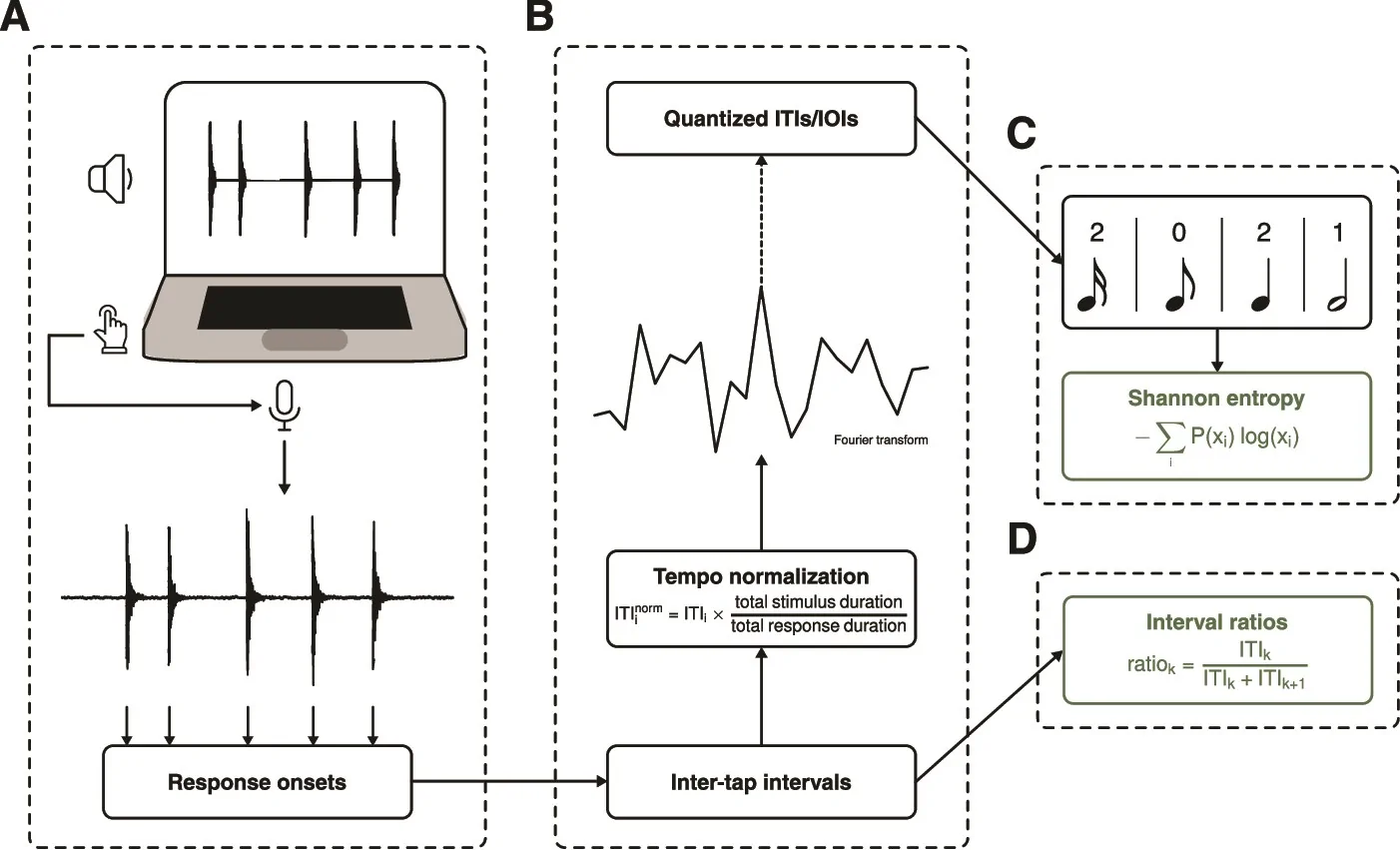
Behavioral setup and analysis pipeline for the rhythm reproduction experiment. A) Participants reproduced the presented random sound sequences by tapping on the casing of the experimental laptop. These taps were recorded via the laptop’s microphone and analyzed using REPP to extract tapping onsets (Anglada-Tort et al. 2022). B) ITIs were derived from extracted tapping onsets and subjected to two parallel data analysis pathways: for interval ratio calculations, raw ITIs were preserved to maintain temporal relationships; for entropy analysis, ITIs underwent tempo normalization to align response duration with stimulus duration, enabling direct comparison between stimulus IOIs and corresponding response intervals. Both normalized response ITIs and stimulus IOIs were subsequently quantized using Fourier transform-based analysis of the respective temporal sequences. C) Quantized ITIs and IOIs were used to calculate Shannon entropy based on the frequencies of occurrence of each note duration. D) Raw response ITIs and stimulus IOIs were the input for calculating interval ratios (Roeske et al. 2020), representing the normalized relationship between the durations of adjacent intervals.

Our neural predictor design, with brain imaging performed days before behavioral testing, allowed testing how intrinsic brain connectivity predicts magnitude and direction of individual transformations of randomly generated rhythms toward more regular ones. While previous investigations have documented musical rhythm’s effects on intrinsic brain architecture (Toiviainen et al. 2020), we reverse this classical approach to show how intrinsic brain architecture shapes musical rhythms. Using a data-driven approach, we specifically tested the hypothesis that rhythmic biases can be predicted by the organization of cortico-subcortical auditory-motor circuits involved in timing and cortical circuits involved in higher-order cognitive processes, while not constraining our search to specific brain networks. Critically, two lines of prior evidence guided these expectations. First, the organization of resting-state functional connectivity is closely linked to higher-order cognitive functions due to its dynamical and flexible nature (Fox and Raichle 2007; Sassenberg et al. 2024) and supports complex musical temporal processes that rely on these general functions (Lumaca et al. 2024). This led us to hypothesize that entropy reduction, a measure of overall rhythmic compressibility, would relate to functional connectivity patterns in those brain circuits supporting higher-order cognitive processes. Second, the well-established involvement of thalamo-basal ganglia-motor cortical circuits in beat-based timing (Grahn and Rowe 2009; Teki et al. 2011; Nozaradan et al. 2017) led us to expect that small-integer-ratio biases (isochrony, binary, and ternary ratios) would relate to the functional and microstructural properties of these pathways. This novel approach may reveal some of the neurobiological boundaries within which cultural variation occurs in music (Lumaca et al. 2018b).

## Materials and methods

### Participants

Data were acquired across two sessions at Aarhus University Hospital from healthy adults. Recruitment criteria included: normal health; age between 18 and 40; normal or corrected-to-normal vision; normal hearing; English speaker; right-handed; no formal musical training. All of them gave their written consent and filled out an MRI safety form. Ethical approval was obtained from De Videnskabsetiske Komitéer for Region Midtjylland, Denmark (case no. 1-10-72-210-22). Of the 49 volunteers (29 females, average age: 26.69 years, range: 18 to 40) who met the selection criteria, 47 completed the experiment: 2 participants (1 female) only took part in the MR session. Participants were nonmusicians, having received no more than 6 years of formal musical training (mean: 1.4 years) (Zhang et al. 2020) (Supplementary Fig. S1).

### Experimental design

Each participant underwent two experimental sessions (MRI and behavioral) on separate days (mean interval: 17.5 d, range 6 to 50; Fig. 1A). After the MRI session (~45 min), they completed WAIS-IV Digit Span/Arithmetic subtests (assessing working memory/attention, ~20 min) and the MET rhythmic subtest (rhythmical perception, ~15 min). The behavioral session involved a rhythm reproduction task, followed by the Gold-MSI musical skills self-report questionnaire.

### MR acquisition

Data were acquired using a 3T Siemens Prisma MRI scanner at the Aarhus University Hospital. During the fMRI resting-state scan (10 min), participants were instructed to keep their eyes closed and refrain from engaging in any specific thought. For each subject, a total of 600 volumes were acquired using fast T2^*^-weighted echo-planar imaging (EPI) multiband sequence (repetition time [TR], 1,000 ms; echo time, [TE], 29.6 ms; voxel size, 2.5 mm^3^). An MP2RAGE sequence was used for the acquisition of a high-resolution T1-weighted (T1w) image (TR = 5,000 ms; TE = 2.87 ms; voxel size = 0.9 mm^3^) (10 min). The HARDI sequence included: 90 diffusion directions at b = 2,800 s/mm^2^; 60 directions at b = 1,200 s/mm^2^; 30 directions, b = 700 s/mm^2^; 11 directions, b = 5 s/mm^2^, with the different b-shells acquired in the same series (flip angle = 90°, TR/TE = 2,972/65 ms, voxel size = 1.8 × 1.8 × 1.8 mm, matrix size = 112 × 112, number of slices = 90). The phase-encoding direction was anterior to posterior (AP). An opposite phase-encoding direction (PA) was also acquired (b = 5 s/mm^2^) to allow EPI distortion correction (Andersson et al. 2003; Holland et al. 2010).

### Behavioral task and stimuli

In the rhythm reproduction task, participants were asked to reproduce a randomly generated, irregular sequence of drum sounds by tapping their index finger on the surface of a laptop. The stimulus was presented first, with participants tapping after sequence presentation. The experiment was conducted in a sound-proof experimental room using a custom website presented on a laptop. The website provided the experiment instructions and controlled the stimulus presentation. Participants were asked to read the instructions carefully before starting the training phase. The training phase was considered completed once participants produced three out of the four training sequences correctly (~1 min). Participants were allowed to repeat the training phase until successful completion. A trial was considered correct if the participant produced the same number of taps as there were sounds in the stimulus. After completing the training phase, participants proceeded to the test phase of the experiment (~30 min).

The stimulus in the rhythm reproduction task was a 70-ms clap sound with a short attack. The test phase employed a 2 × 2 within-subject design: stimulus sequences were either slow (600 ms mean inter-onset intervals, IOIs) or fast (400 ms mean IOI), and comprised either four or five presented sounds. This design provided graded task difficulty, ranging from easiest (fast, four sounds) to most difficult (slow, five sounds) (Supplementary Fig. S2). A total of 192 trials were presented with an equal number of trials for each of the four conditions. Sequences were generated individually for each participant by randomly sampling IOIs (van der Werff et al. 2025) from a uniform distribution with boundaries 350 and 850 ms (slow trials) or 150 and 650 ms (fast trials). To reduce proximity to isochrony, IOIs within ±120 ms (slow) or ±80 ms (fast) of the distribution mean were excluded. The experiment comprised four blocks with 1-min breaks in between. To avoid carry-over effects, slow and fast trials alternated, and trial lengths were randomized across the session.

### Analysis of finger-tapping data

Finger-tapping data were analyzed using Rhythm ExPeriment Platform (REPP) (Anglada-Tort et al. 2022), which extracts tapping onsets from audio recordings. To calculate Shannon entropy and edit distance (but not interval ratios; Fig. 2), responses were tempo-normalized by scaling all inter-tap intervals (ITIs) by the ratio of total stimulus duration to total response duration, preserving relative durational patterns while removing global tempo differences. Subsequently, both response and stimulus onsets were quantized using a custom Python script. Following Ravignani and Norton 2017, a Fourier transform of the sequence represented as zeros and ones (temporal resolution = 1 ms) identified the dominant periodicity, defining the fundamental grid unit (a “sixteenth note,” approximately one-quarter of the mean beat duration). Each continuous interval was then rounded to the nearest integer multiple of this grid unit, yielding discrete quantized durations.

#### Shannon entropy

Shannon entropy (H) represents the amount of information in a signal, where lower entropy means a smaller amount of information (ie larger predictability), and higher entropy means a larger amount of information (ie lower predictability; Shannon 1948; Lumaca and Baggio 2016; Ravignani and Norton 2017; Lumaca et al. 2018a; Lumaca et al. 2019a; Lumaca et al. 2019b). Here, we calculated H for tempo-normalized and quantized note durations (IOIs for stimulus; ITIs for response) using the following equation:


$$ H=-\sum{p}_x lo{g}_2{p}_x $$

where ${p}_x$ is the probability that the symbol in the signal (ie quantized note duration) occurs in that sequence. Entropy is maximal when all note durations occur with equal probability (ie maximum variability) and minimal when a single duration repeats throughout (isochrony). To quantify regularization, we computed the change in entropy from stimulus to response for each trial. By averaging across trials, this yielded a single value per participant. Negative values indicate regularization: participants reproduced rhythms as more predictable sequences than the original stimuli. Although entropy is computed on quantized data, the quantization step does not predetermine the entropy outcome. Quantization maps continuous interval durations onto a shared categorical scale (multiples of a sixteenth note), but participants who distribute intervals across many duration categories still produce high entropy, while those who cluster around a few categories produce low entropy.

#### Simple interval ratios

Following Roeske et al. (2020), interval ratios were calculated as the normalized ratio between consecutive IOIs ($r_k = \frac{\textrm{IOI}_k}{(\textrm{IOI}_k + \textrm{IOI}_{k+1}}$). The resulting distribution was divided into equal-sized bins whose boundaries, though historically determined quasi-arbitrarily, have recently received perceptual support (Roeske et al. 2020; De Gregorio et al. 2021; Barbero et al. 2025). Bin centers represent, in alternation, ratios expressible as small integers (1, 2, 3) and ratios that are not. As an example, the bin center of $r_k = 0.33$ can be described using small integers, because if we imagine two intervals where the second interval is twice as long as the first interval (ie a 1:2 relationship between intervals) $r_k = \frac{1}{(1 + 2)} = 0.33$. However, the bin centered around ($r_k = 0.29$ cannot be described using small integers. We only considered the small integers 1, 2, and 3. A ratio was classified as isochronous if falling into a 1:1 relationship between consecutive intervals (bin center, $r_k=0.5$), binary if falling into the 1:2 or a 2:1 relationship (bin centers, $r_k=0.33$ and $r_k = 0.66$), and ternary if falling into the 1:3 or 3:1 relationship (bin centers, $r_k = 0.25$ and $r_k = 0.75$). For each participant and condition, bin counts were normalized by the total number of ratios. To account for the differently sized bins, the number of observations within a bin was divided by the total number of observations (ie all ratios within a condition, within participant). Finally, the number of isochronous, binary, and ternary ratios introduced or removed in the response relative to the stimulus was computed by subtraction, yielding indices of spontaneous regularization toward small-integer ratios.

#### Edit distance

Edit distance (Levenshtein distance) between stimulus and response was calculated on tempo-normalized and quantized responses using the Python package *thebeat* (van der Werff et al. 2024). As implemented here, edit distance is an integer value used to describe the similarity between the stimulus and the response; it was used here as a global measure of reproduction error to identify which experimental condition elicited the largest deviations from the stimulus. To calculate it, after quantization, we first converted each stimulus and response sequence into a series of zeros and ones. The length of this series was given by the total duration of the sequence divided by the duration of the “sixteenth note” resulting from the Fourier transform. Each zero represents a metrical grid unit, and each one represents the onset of a sound or tap. Seeing as for each stimulus–response pair, both series were always of equal length (because of the tempo normalization procedure), the edit distance thus represents the sum of the number of substitutions that are required to transform the stimulus series into the response series. A larger edit distance indicates higher reproduction error, reflecting greater dissimilarity between the stimulus and the response. Conversely, a smaller edit distance suggests a more accurate reproduction. An edit distance of 1 would mean that for one of the stimulus onsets, the participant reproduced it such that it occurred in a different place in the metrical grid, which in turn results in two changed intervals. Here, the maximum edit distance for a sequence of four sounds is 2 (three changed intervals), and for a sequence of five sounds 3 (four changed intervals).

A repeated measures ANOVA analysis was employed to examine the effects of tempo (400 vs. 600 ms mean IOI) and sequence length (4 vs. 5 sounds) on rhythmic behavior. Across the regularization measures (entropy, isochrony, binary, or ternary ratios), no significant main effects of tempo or length were observed, nor significant interaction effects between these factors (Supplementary Tables S2 and S3). All behavioral scores were therefore aggregated across experimental conditions for each participant. Subsequently, for each participant, individual summary scores were calculated. For entropy and edit distance, this was the mean of the participant’s scores. For the number of isochronous and the number of binary/ternary ratios introduced, this was the total number of observed ratios in the respective bin(s) divided by the total number of recorded ratios for that participant.

### Preprocessing of resting-state fMRI

Functional connectomes were generated from rs-fMRI images by using the default surface-based preprocessing pipeline from the CONN toolbox release 22.a (RRID:SCR_009550) (Whitfield-Gabrieli and Nieto-Castanon 2012) and SPM (RRID:SCR_007037) release 12.7771 (Penny et al. 2011), implemented in Matlab 2016b (The MathWorks, Inc., Natick, MA, USA) (Fig. 3A). Subject-level preprocessing included: realignment with motion correction; SPM realign and unwarp procedure (Andersson et al. 2001); coregistration of all scans to a reference image (first scan of the first session) using a least squares approach and a 6-parameter (rigid body) transformation (Friston et al. 1995); resampling using b-spline interpolation to correct for motion and magnetic susceptibility interactions; identification of potential outlier scans using ART (Whitfield-Gabrieli et al. 2011) as acquisitions with framewise displacement above 0.9 mm or global blood-oxygen-level-dependent (BOLD) signal changes above 5 standard deviations (Whitfield-Gabrieli et al. 2011; Power et al. 2014); computation of a reference BOLD image for each subject by averaging all scans excluding outliers; coregistration of functional and anatomical data using the SPM intermodality coregistration procedure (Ashburner and Friston 1997) with a normalized mutual information objective function. Functional data were resampled to the FreeSurfer fsaverage cortical surface (Fischl 2012) and smoothed with an 8 mm full width at half maximum (FWHM) kernel (Hagler Jr et al. 2006). Denoising of functional data using a standard denoising pipeline (Nieto-Castanon 2020a), including regression of 5 white-matter and 5 CSF noise components (CompCor; Chai et al. 2012), 12 motion parameters with their first-order derivatives (Friston et al. 1996), outlier scan regressors (Power et al. 2014), session effects, and linear trends, followed by bandpass filtering (0.008 to 0.09 Hz). CompCor has been shown to preserve biological anti-correlations while controlling for motion and physiological artifacts (Chai et al. 2012). Subject-wise symmetric connectivity matrices were then constructed across 68 cortical and 16 subcortical nodes of the Desikan-Killiany atlas (84 × 84 functional connectivity matrices; Desikan et al. 2006), with each edge represented by Fisher z-transformed bivariate correlation coefficients derived from a weighted general linear model (Nieto-Castanon 2020b).

**Figure 3:**
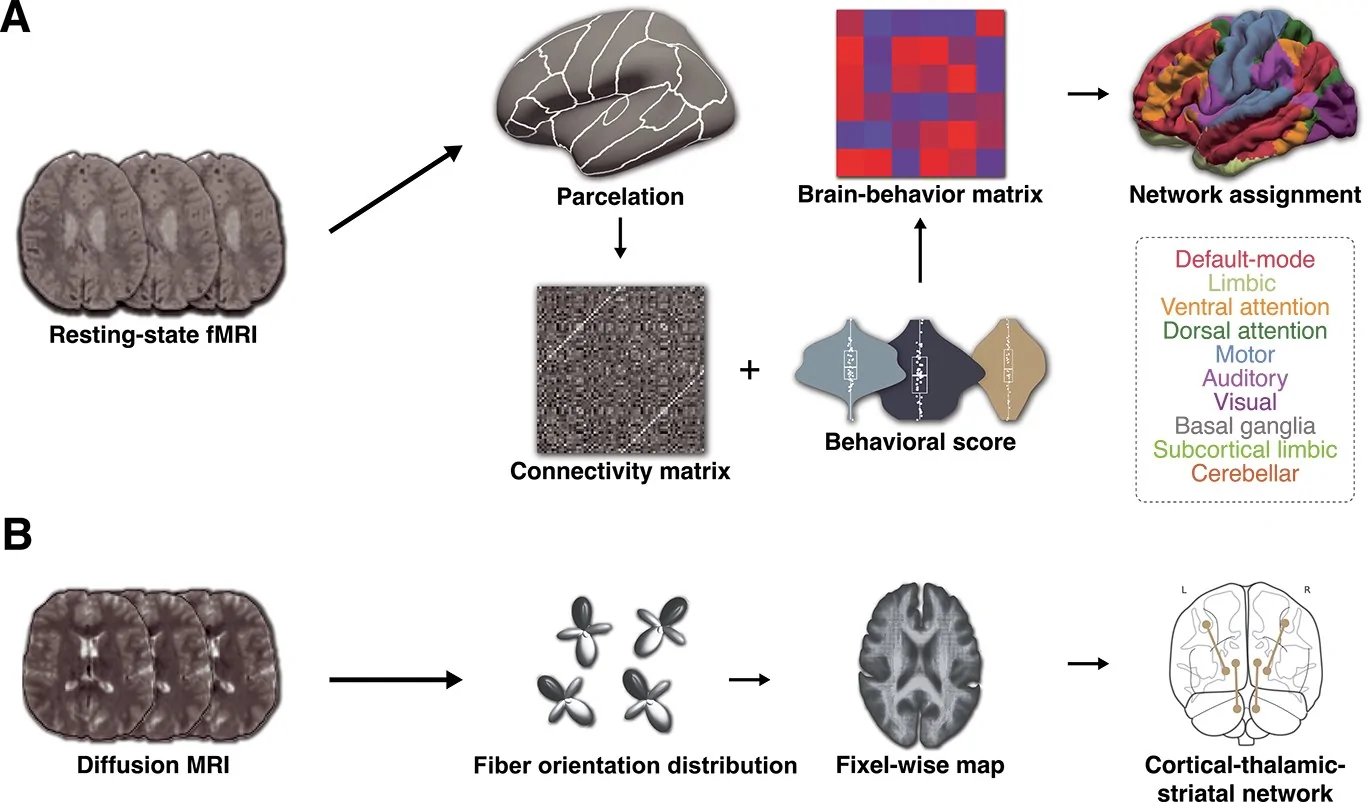
Schematic overview of the resting-state fMRI and dMRI analysis pipelines. A) CPM of resting-state fMRI data with 5-fold cross-validation. To aid functional interpretations, the resulting brain connectomes were assigned to 10 canonical resting-state networks. B) Fixel-based analyses of diffusion MRI (dMRI) data with a schematic representation of the motor cortic0-subcortical and cerebellar networks.

### Connectome-based predictive modeling of resting-state functional data

Functional connectivity matrices were entered into connectome-based predictive modeling (CPM) (Shen et al. 2017) to build neuro-predictive models of rhythmic behaviors. In this analysis, we implemented a robust 5-fold cross-validation approach with 50 iterations to ensure stable predictions across behavioral traits (stimulus–response entropy difference, binary/ternary ratios, isochrony). Participants were randomly partitioned into training and testing folds across different configurations to capture full predictive stability. Within training folds, Spearman correlations between edge connectivity and behavioral scores established association matrices, with significant edges (*P* < 0.01) forming positive networks (for positive correlations) and negative networks (negative correlations). A stringent consensus approach retained only edges always appearing across all 50 repetitions, ensuring maximum network stability and generalizability. For each participant and network type, supra-threshold edges were summarized into subject-wise connection strength scores reflecting the aggregate network connectivity’s influence on individual behavioral performance. These network strength vectors were used in linear regression as predictors, their parameters fitted on training data to predict behavioral outcomes in test participants. Final predictions were averaged across all iterations to reduce variance and enhance reliability. Model performance was evaluated using Pearson correlations between predicted and actual values, with statistical significance assessed via nonparametric permutation testing (*n* = 1,000, p_perm  < 0.05).

To delineate the functional neuroanatomy of the predictive networks, Desikan cortical parcels were allocated to ten large-scale resting-state networks (Shirer et al. 2012), comprising several cortical (Default Mode Network, Ventral Attention Network, Auditory Network, Visual Network, Motor Network, Dorsal Attention Network, and Limbic Network) and three subcortical networks (Basal Ganglia, Limbic Subcortical, and Cerebellar) (Supplementary Table S4). This parcellation strategy merged functionally related regions from the Shirer’s atlas, such as dorsal/ventral default mode and anterior/posterior salience, to create coherent network units representing major brain systems. Between-network connectivity was assessed through degree-based analysis of cross-validated predictive networks. Weighted network matrices were binarized to identify edges connecting Desikan regions, with inter-network connection sums calculated for each network pair. This approach quantifies the functional coupling between distinct brain systems. Statistical significance was determined via nonparametric permutation testing (*n* = 1,000), where null distributions were generated by randomly shuffling node-to-network mappings and recalculating inter-network connections. As network size was preserved in permutations, the null distribution for each network pair was inherently scaled to those sizes, obviating the need for explicit normalization of connection counts. Resulting *P*-values underwent Benjamini–Hochberg false discovery rate (FDR) correction (q = 0.05) to control for multiple comparisons across all network pairs, ensuring robust identification of significant inter-network relationships.

### Preprocessing of diffusion data

Diffusion data were processed using the fixel-based analysis (FBA) pipeline (https://www.mrtrix.org) in MRtrix3 (Version 3.0_RC3) (Tournier et al. 2019) (Fig. 3B). Preprocessing included MP-PCA denoising (Veraart et al. 2016), Gibbs ringing artifact removal (Kellner et al. 2016), eddy current, motion, and susceptibility-induced distortion correction using the reverse phase-encoding acquisition (Andersson et al. 2003; Andersson and Sotiropoulos 2016), and spatial intensity inhomogeneity correction (Tustison et al. 2010). Image quality was assessed via the FSL Quality Assessment for dMRI (QUAD) and Study-wise Quality Assessment for dMRI (SQUAD) (Bastiani et al. 2019); two participants were excluded based on signal-to-noise ratio and head motion criteria (>2 SD from mean), yielding *n* = 45 for all fixel-based analyses. Next, an automated (unsupervised) approach (“dhollander”) was used on preprocessed diffusion-weighted images to obtain three-tissue response functions representing single-fiber white matter, gray matter, and cerebrospinal fluid (Dhollander et al. 2016), then averaged across participants. Diffusion MRI data were upsampled to an isotropic voxel size of 1.3 mm^3^ using the (default) cubic interpolation method to increase anatomical contrast and improve the downstream generation of a population template, and a brain mask was estimated. Fiber orientation distributions (FODs) were estimated using multi-shell multi-tissue constrained spherical deconvolution (MSMT-CSD) using the three average tissue response functions (Tournier et al. 2007). Further processing steps included: global intensity normalization and joint bias field correction of three-tissue compartment parameters (Raffelt et al. 2017a) and the generation of a study-specific FOD population template from the intensity-normalized white-matter FODs of all 45 participants (Raffelt et al. 2011).

We then followed the pipeline outlined in Fuelscher et al. (2021) to produce fixel-based metrics in MNI space. Specifically, we first co-registered the study-specific population template to MNI space using fractional anisotropy-based affine registration in FSL. Next, we registered the participants’ FOD images to the new template space and segmented them into discrete fixels (Smith et al. 2012; Fuelscher et al. 2021).

### Fixel-based analysis of diffusion-weighted data

Three fixel-based metrics of white-matter microstructure were calculated in every white-matter fixel for each subject: fiber density (FD), a microstructural measure reflecting the intra-axonal volume fraction within a fiber population and thus the degree of axonal packing (Raffelt et al. 2012; Genc et al. 2020); logarithmic-transformed fiber cross-section (log FC), a macrostructural measure related to the cross-sectional area of fiber bundles (Raffelt et al. 2012); and fiber density and cross-section (FDC), the product of FD and FC, related to the total estimated intra-axonal volume and sensitive to both micro- and macrostructure. Once these metrics were computed, fixel directions were reoriented to correct for spatial normalization, and each subject’s fixels were matched to the corresponding fixel in the template image to enable group-level statistical comparisons (Raffelt et al. 2017b). FBA overcomes the partial volume effects that limit voxel-based methods by resolving individual fiber populations within a single voxel (Jeurissen et al. 2014; Dhollander et al. 2021).

Using TractSeg (Wasserthal et al. 2018)—a semi-automated method of white matter dissection based on deep neural networks—we delineated 5 bilateral tracts of interest (Supplementary Fig. S7) from the 72 available. These tracts constitute the major white-matter pathways of two systems implicated in rhythm processing: a cortical motor-subcortical network (comprising thalamus- and striato-precentral, and thalamus- and striato-premotor tracts) for beat-based timing (Grahn and Brett 2007; Teki et al. 2011; Schwartze and Kotz 2013; Merchant et al. 2015) and its connections to the cerebellar network (via inferior, middle, and superior cerebellar peduncles) for duration-based timing (Grube et al. 2010; Schwartze and Kotz 2013). The 5 tracts included: cerebellar peduncles (CP; inferior, middle, superior), connecting cerebral cortex and cerebellum via thalamus and pontine nuclei; striato-premotor (ST-PREM) and striato-precentral (ST-PREC), connecting putamen to premotor and primary motor cortices, respectively, providing a direct pathway from basal ganglia to motor planning and execution areas; and thalamo-premotor (T-PREM) and thalamo-precentral (T-PREC), connecting ventral thalamus to premotor cortex/supplementary motor area and primary motor cortex, respectively. These tracts provide the structural substrate for the functional interactions between the basal ganglia-thalamic, motor, and cerebellar networks examined in our CPM analyses (Kasdan et al. 2022). For each participant and tract, fixel metrics (FD, log FC, FDC) were averaged across all fixels assigned to that tract, with bilateral tracts averaged across hemispheres to yield a single value per tract.

A general linear model (GLM) with ordinary least squares regression investigated the relationship between each behavioral measure (dependent variable) and fixel-based metrics (FD, log FC, FDC; predictors) in bilateral white matter tracts. The GLM (Y = β₀ + β₁X₁ + β₂X₂ + β₃X₃ + ε; Y = behavioral, X₁ = fixel metrics) included covariates for relative head motion (X₂  = average frame-wise displacement) (Bastiani et al. 2019) and log-transformed total intracranial volume (X₃ = eITV). eITV, calculated with FreeSurfer from T1 anatomical images, was only applied to regressions including log FC and FDC as predictors, to mitigate template registration brain scaling effects (Smith et al. 2012). *P*-values per fixel metric were Benjamini–Hochberg FDR-corrected (q = 0.05) for 15 independent tests (5 tracts ×3 behavioral metrics).

## Results

### Behavioral signatures of rhythmic biases

Participants showed systematic biases when reproducing the random rhythmic sequences (Fig. 4). Overall, they reduced entropy in the reproduced sequences (participant median: −0.08; range: −0.30 to 0.12; Wilcoxon signed-rank test *P* < 0.001; Wilcoxon effect size r = 0.718, two-sided) and increased the occurrence of binary and ternary ratios (participant median: 0.06; range: −0.03 to 0.18; Wilcoxon signed-rank test *P* < 0.001; Wilcoxon effect size r = 0.793, two-sided). These are robust effects, with considerable inter-individual variation. Notably, there was no significant group preference for introducing isochrony (median: −0.002; range: −0.14 to 0.13; *P* = 0.773; r = 0.0432), with approximately half of the participants (48.9%) increasing it, while the other half (51.1%) decreasing it. Half of the participants showed a negative bias toward isochronous intervals, but a strong positive bias toward 1:2 and 1:3 ratios. This suggests the presence of competing attractor dynamics in rhythmic timing. Specifically, these dynamics may reflect a competition between beat-based timing (favoring isochronous, metronome-like intervals) and duration-based timing (favoring hierarchical structures based on small integer ratios). This pattern aligns with previous findings by Repp and colleagues (Repp et al. 2012a, 2012b). Supporting this interpretation, we found a significant negative correlation between isochrony and binary/ternary ratios: participants with lower isochrony tended to show a stronger bias toward 1:2 and 1:3 ratios (r = −0.62, pFDR < 0.001) (Supplementary Fig. S3). In part, this negative correlation is to be expected (fewer isochronous ratios may mean more binary/ternary ratios). However, these measures are not fully orthogonal: it is very possible for a participant to reproduce fewer isochronous ratios than were present in the stimulus, without introducing binary and/or ternary ratios (and vice versa).

**Figure 4:**
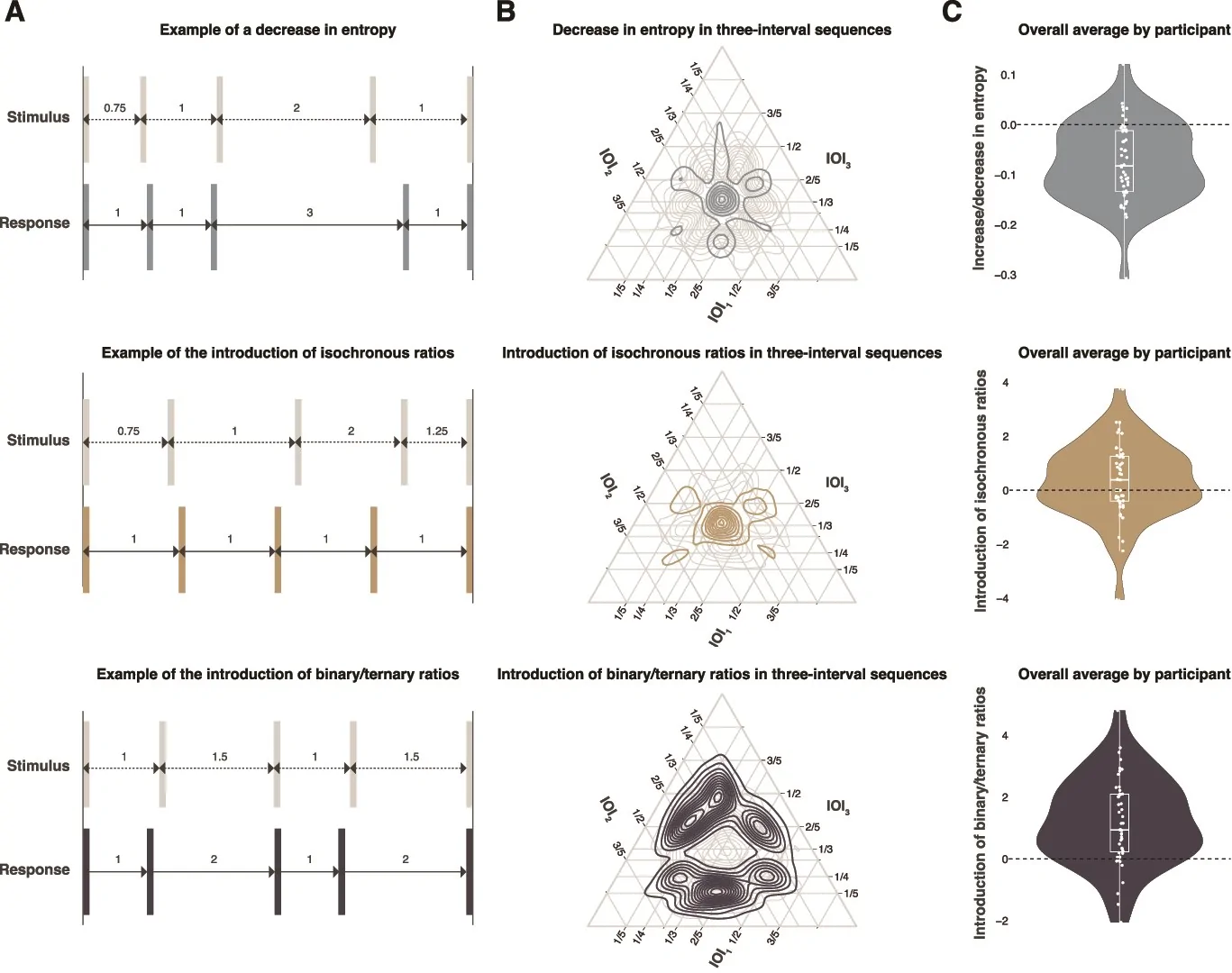
Behavioral results from the tapping reproduction task. A) Exemplary stimulus–response pairs observed for each of the three behavioral measures used in the analyses. The absolute numerical values are arbitrary, representing the intervals’ relative duration to each other. A decrease in entropy (top) is the result of the clustering of interval durations around few values: while the stimulus contained three durational values, the response only contained two. An increase in the number of isochronous ratios (middle) indicates that, in the response, the participant introduced equal-duration pairs of adjacent intervals that were not present in the initial stimulus. An increase in the number of binary or ternary ratios (bottom) indicates the introduction of pairs of adjacent intervals that have a binary (the second interval in the pair is twice as long as the first) or ternary (the second interval in the pair is three times as long as the first) relationship to each other. B) Ternary plots of all observed tapping responses to four-sound (ie three-interval) sequences that contained a decrease in entropy (entropy difference in the response compared to the stimulus < 0), an increase in isochronous ratios (number of isochronous ratios in the response compared to the stimulus > 1), and an increase in binary or ternary ratios (number of binary/ternary ratios in the response compared to the stimulus > 1). Note that only four-sound sequences are represented here; the other half of the trials contained five sounds. Any point in a ternary plot represents the relative durations of three intervals, normalized such that the intervals sum to 1. For example, a point in the plot may represent relative, consecutive interval durations of one-fifth, three-fifths, and one-fifth. Stimulus sequences are represented in the background of the ternary plots in light gray. C) Violin plots show that during tapping reproduction, on average, participants decreased information content (lower entropy), did not increase the amount of isochrony, yet introduced binary and ternary ratios. Each point in the plot represents the averaged result for a participant (*n* = 47) across all trials.

The general tendency toward more regular patterns showed substantial individual variation. While individual differences in rhythmic abilities among nonmusicians are well-documented in music research (Müllensiefen et al. 2013; Correia et al. 2023), they have been largely neglected in iterated and serial reproduction experiments. Computational models of iterated learning suggest that these differences may be critical for understanding how regularization behaviors shape cultural systems, with some features reflecting the extreme biases of certain individuals (Navarro et al. 2018). The observed variation in individual biases for rhythmic regularities, coupled with an overall tendency toward structured patterns, suggests that these biases might stem from individual variability in intrinsic neural circuitry (Waschke et al. 2021), including both brain function and brain structure (Kanai and Rees 2011; Harmelech and Malach 2013). To test this hypothesis, we examined the neural bases of rhythmic transmission biases focusing on the organization of resting-state functional networks—patterns of coordinated activity between brain regions emerging from the resting brain—and microstructural features of white matter pathways involved in rhythmic perception and memory.

### Variability in functional network interactions predicts entropy reduction

We employed CPM (Shen et al. 2017)—a data-driven framework that constructs neuropredictive models from individual connectivity patterns—to examine whether intrinsic functional network organization predicts rhythmic regularization biases. CPM identifies connectivity features (edges) that correlate with behavioral measures in training data, then generates “predicted” behavioral scores for unseen participants based on their connectivity profiles, with model performance assessed through k-fold cross-validation. This method represents a higher-level predictive modeling approach than within-sample “correlations” (Gabrieli et al. 2015), yielding realistic estimates of generalizability. In our study, the data input for CPM were functional connectomes: the comprehensive map of resting-state functional connections (edges) across brain regions (nodes). Functional connectomes were constructed from resting-state fMRI using cortical areas of the Desikan-Killiany atlas (Desikan et al. 2006) and subcortical segmentations, for a total of 84 nodes. These connectomes yielded 3,486 possible edges per participant. To facilitate neuroanatomical interpretation, these nodes were assigned to 10 canonical large-scale networks (Supplementary Table S4*)*: 7 were cortical (limbic, ventral and dorsal attention, motor, auditory, visual, and default mode) and 3 subcortical (basal ganglia, subcortical limbic, and cerebellar) (Finn et al. 2015). This hierarchical organization in the processing pipeline enabled examination of first edge-level connectivity, and then between-network functional coupling patterns, underlying individual behavioral differences.

Results are shown in Fig. 5. Correlating predicted and observed values (Pearson), we found that CPM with 5-fold cross-validation significantly predicted the stimulus–response decrease of rhythmic entropy in novel individuals (r_pred-obs_ = 0.38; p_perm = 0.01; Supplementary Fig. S4B). A larger entropy decrease was associated with increased strength within a “negative” CPM network (ie a set of edges negatively correlated with entropy change) (Fig. 5D). The negative network consisted of 27 edges (0.77% of the 3,486 total possible edges) (Fig. 5A and C). This network was predominantly bilateral (19 interhemispheric edges; Supplementary Table S5). Network topology analysis revealed that between-network edge counts for two network pairs exceeded chance expectations based on nonparametric permutation testing (*n* = 1,000 permutations shuffling node-to-network assignments): dorsal attention network (DAN) to default mode network (DMN) connections (10 edges; Z = 5.55, pFDR < 0.001) and basal ganglia-thalamus (BG-TH) to auditory network (AN) connections (4 edges; Z = 5.35, pFDR = 0.01; Fig. 5B and E; Supplementary Table S6). DAN-DMN edges predominantly linked left-hemisphere DAN nodes (Pars Orbitalis, Pars Triangularis, Pars Opercularis) with right-hemisphere DMN nodes (Inferior Parietal cortex, Medial Orbital Frontal cortex, Precuneus, Rostral Anterior Cingulate cortex). Similarly, the AUD-BG edges predominantly pair left auditory regions (Banks superior temporal sulcus, Superior temporal gyrus) with right basal ganglia structures (Putamen, Thalamus). Participants exhibiting greater entropy reduction displayed reduced anti-correlation between DAN and DMN, alongside increased BG-TH-AN functional coupling (Supplementary Fig. S5; participants split at median entropy change for visualization). These results were replicated using 10-fold cross-validation (k = 10; r = 0.36, p_perm = 0.03; Supplementary Fig. S6; Supplementary Tables S7 and S8). No significant relationships emerged for isochronous interval changes or binary/ternary ratio changes.

**Figure 5:**
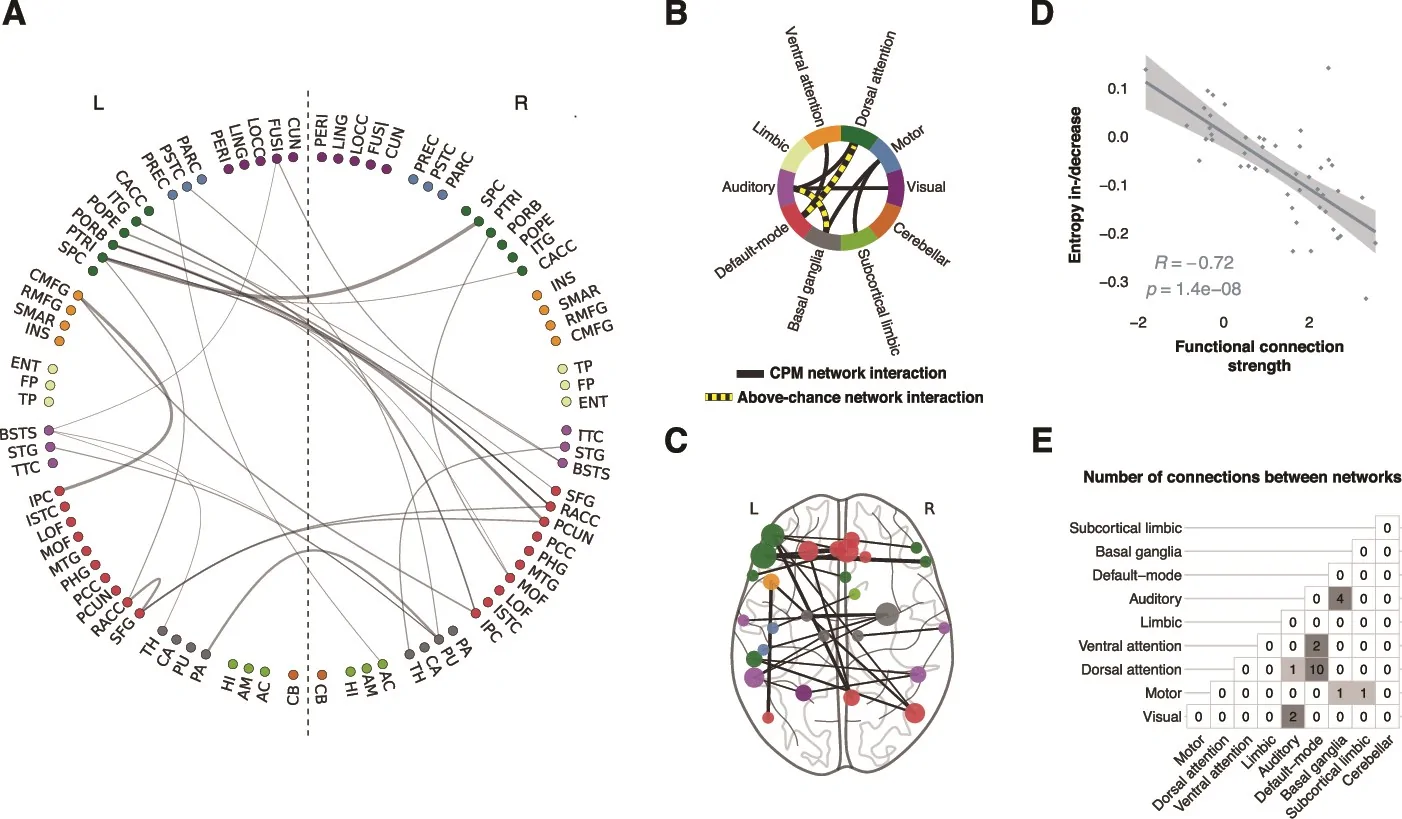
Results from CPM. A) Functional connectivity patterns of the negative model network predicting response-minus-stimulus changes in rhythmic entropy using a robust 5-fold cross-validation. The circular connectogram represents the distribution of edges between brain regions. Nodes (spheres) are color-coded by network affiliation: visual (deep purple), motor (steel blue), dorsal attention (dark green), ventral attention (orange), limbic (light green), auditory (magenta), default mode (red), basal ganglia-thalamic (gray), sub-limbic (soft green), and cerebellar (warm orange). The strength of the connections is indicated by the width of the lines. B) Negative predictive network displayed as interactions between large-scale resting-state networks. Dotted (yellow) lines in the circular connectogram indicate between-network connections whose count is statistically significant after nonparametric permutation statistics (*n* = 1,000) and FDR correction for multiple comparisons. C) Glass brain plot displaying the negative predictive network: nodes (spheres) are sized by their degree (number of edges). Node colors reflect the 10-network assignment. D) The decrease in rhythmic entropy is significantly predicted by functional interactions between auditory and basal ganglia networks, and between dorsal attention (task-positive) and default mode network (task-negative). Functional connection strength represents the aggregate CPM-derived network score for each participant, computed as the sum of edge weights (Fisher z-transformed correlation coefficients) across all edges in the negative predictive network. Each data point in the scatterplot represents one participant (*n* = 47). E) Between-network connections count matrix. Cells indicate the number of connections between network pairs; darker colors represent a larger number of connections.

### Variability in the microstructure of physical pathways is associated with isochrony biases

In a separate analysis, we examined whether individual differences in rhythmic biases were also reflected in the physical organization of brain connections using diffusion MRI (Fig. 6), which measures how water molecules move along white matter pathways (Le Bihan et al. 2001). Using FBA (Tournier et al. 2007)—a technique that provides fiber-specific measurements of diffusion MRI data—we focused on five bilateral tracts that belong to two interfacing systems, the striatal-thalamic-motor cortical and the cerebellar networks, both involved in rhythmic perception and production (Kasdan et al. 2022): the cerebellar peduncle (CP), striato-premotor (ST-PREM), striato-precentral (ST-PREC), thalamo-premotor (T-PREM), and thalamo-precentral (T-PREC) tracts (Wasserthal et al. 2018). For each tract, we quantified (i) the average FD, reflecting the degree of axonal packing in a fiber population; (ii) fiber cross-section (log10 FC), indicating the cross-sectional size of a fiber population; and (iii) a combination of these two metrics (FDC) (Raffelt et al. 2017b) (Supplementary Fig. S7).

**Figure 6:**
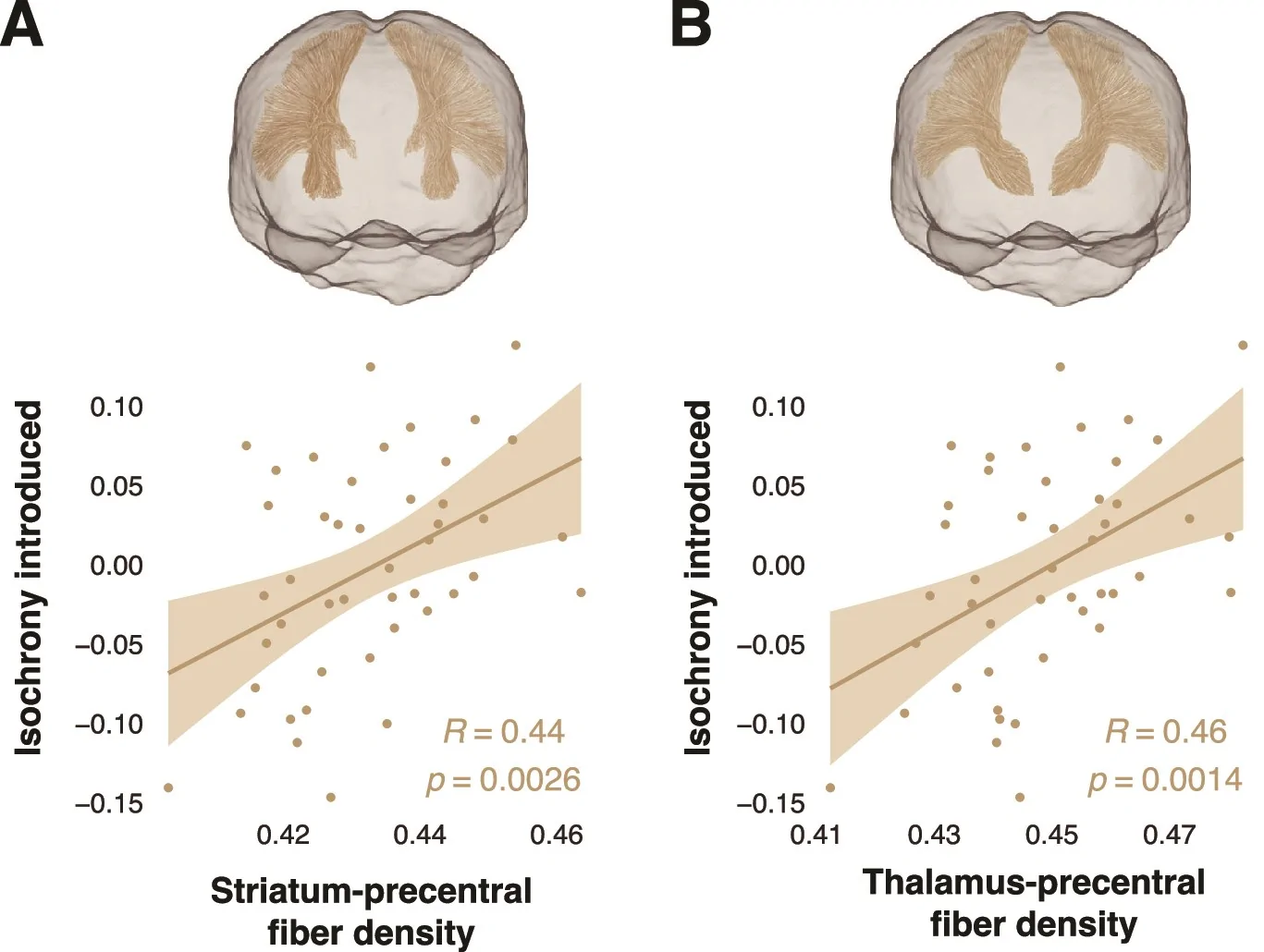
Fixel-based analyses of diffusion-weighted imaging data. A) Participants who introduced more isochronous ratios than those present in the stimulus via their finger-tapping response display larger fiber density in striatum-precentral white-matter bundles. B) Similarly, participants who introduced isochrony also display larger fiber density in thalamus-precentral white-matter bundles. In both panels, each data point in the scatterplot represents one participant (*n* = 45).

Multiple linear regression revealed that the tendency to produce isochronous intervals positively correlated with fiber density in two key subcortical–cortical motor pathways: the striato-precentral tract (Fig. 6A; r = 0.44, pFDR = 0.02) and the thalamus-precentral tract (Fig. 6B; r = 0.46, pFDR = 0.02) (Supplementary Table S9). Denser subcortical–cortical motor pathways biased the production of metronomic-like rhythmic patterns. No other relationships between tract properties and rhythmic behaviors survived correction for multiple comparisons (Supplementary Tables S10 and S11).

## Discussion

Our findings show that intrinsic brain network organization is statistically associated with biases toward regularization of temporal patterns during rhythm reproduction. We found that the tendency to decrease entropy in complex rhythms is associated with brain function, specifically with cross-network interactions supporting rhythm perception (basal-ganglia-thalamic-auditory connectivity) and executive functions (task-positive/negative interactions). Further, the increase of rhythmic isochrony was statistically associated with white-matter microstructure, namely greater fiber density in a cortico-basal ganglia-thalamo-cortical loop supporting beat-based timing mechanisms. This dissociation suggests that functional connectivity captures aspects of regularization linked to working memory, such as the tendency to decrease information content in rhythmic material, while structural connectivity mainly predicts aspects of regularization related to the sensory components of rhythmic material, including rhythmic categories.

The functional connectivity architecture predictive of entropy reduction points to a specific neurophysiological mechanism. The DAN and DMN are intrinsically anti-correlated (Fox et al. 2005), and weaker anti-correlation has been associated with greater behavioral variability, a marker of less efficient attentional control (Kelly et al. 2008), potentially reflecting inefficient resource allocation between externally and internally directed processing. Although our study design does not directly capture attentional mechanisms in a rhythm task, this pattern is consistent with a framework where diminished segregation between these networks, a marker of reduced shifting from self-referential to task-related processing (Raichle 2015) and associated with impaired cognitive control (Anticevic et al. 2012), compromises faithful stimulus encoding and maintenance of complex temporal patterns in working memory. Such a functional bottleneck could increase reliance on internal timing generators, prompting individuals to impose structure on less faithfully encoded stimulus representations. Concurrently, increased BG-TH-AN connectivity indicates greater integration within the neural circuit that supports temporal prediction (together with cerebellar system) and rhythmic structure extraction (Grahn and Rowe 2009; Sitek et al. 2022). When attentional resources are reduced (ie when DMN and DAN are less segregated), the intrinsic biases of the BG-TH-AN circuit toward regular, predictable temporal patterns may exert a disproportionate influence on motor output. Specifically, oscillatory activity in the beta-frequency band within the basal ganglia-thalamic circuits, coupled with delta/theta-band entrainment in auditory cortex (Morillon et al. 2014), generates temporal predictions that assign greater weight to rhythmic priors, thereby leading to regularization of complex input toward simpler categorical structures. This dual mechanism—reduced executive control combined with increased engagement of timing circuit—provides a systems-level account for individual differences in a tendency to compress temporal information toward lower entropy configurations. Converging evidence from the Action Simulation for Auditory Predictions (ASAP) model (Cannon and Patel 2021), neuropathological studies (Schwartze et al. 2011), and electrophysiological work (Nozaradan et al. 2012) supports the idea that basal ganglia activity is key to temporal structuring.

These results on entropy reduction are consistent with our initial hypothesis that rhythmic regularization relates to the functional organization of brain circuits supporting higher-order cognitive function. Entropy reduction reflects the domain-general cognitive principle of “compressibility” (Rissanen 1978), enabling the most efficient encoding of a serial input, increasing predictability and reducing memory demands (Chater 1999). Previous cultural transmission experiments show that this principle operates during intergenerational transmission, steering artificial cultural material toward compressible, easy-to-learn forms that embed core properties of cultural systems (Kirby et al. 2015; Tamariz and Kirby 2015; Lumaca et al. 2021). An isochronous sequence, with zero entropy, exemplifies the most efficient representation where only a single interval duration needs to be stored and repeated (Ravignani and Madison 2017; Coissac et al. 2026). On the other hand, low entropic sequences can differ from an isochronous organization: for example, alternating patterns of two or three duration categories achieve high compressibility through categorical reduction rather than temporal isochrony. Our finding, consistent with other single reproduction work showing a tendency to equalize intervals within a tone sequence (Povel 1981; Jacoby and McDermott 2017), suggests that intrinsic brain function may underlie this phenomenon. Resting-state functional connectivity is strongly linked to higher-level cognitive functions like working memory, which require complex and temporally integrated processing (Lumaca et al. 2024). This suggests that the cultural evolution of temporal predictability in music, reflecting the amplification of compression biases through serial reproduction experiments (Ravignani et al. 2016; Lumaca and Baggio 2017; Anglada-Tort et al. 2023), may be primarily influenced by a functional bottleneck in information processing. Critically, a result which we did not predict a priori is that pressures for compressibility co-occur with interactions within basal-ganglia-thalamic-auditory networks, which are thought to provide the neural basis for sequence regularization. The observed lateralization patterns in the CPM network provide a further level of interpretation. Left-hemispheric regions, including inferior frontal gyrus (IFG), an area specialized for temporal hierarchy processing (Fitch and Martins 2014; Heard and Lee 2020), and auditory cortex, which preferentially resolves rapid temporal modulations (Poeppel 2003), may favor encoding and reproduction of metrically organized sequences. The right-lateralized basal ganglia–thalamic nodes identified in our CPM model might carry the most predictive variance from bilateral subcortical timing circuits that generate beat predictions (Grahn and Rowe 2009; Kotz and Schwartze 2011), potentially entraining motor output toward low-entropic sequences during reproduction.

The absence of group-level isochrony bias, as found in previous iterated learning work (Ravignani et al. 2016), warrants cautious interpretation. Firstly, it may reflect competing attractor dynamics in interval timing. Previous rhythmic reproduction work (Repp et al. 2012b) demonstrates that temporal intervals are subject to dual regularization forces: assimilation toward isochrony (1:1) and toward small integer ratios (1:2, 1:3), with neural attractors positioned between these targets. When stimuli sample randomly across both attractor basins, participants produce responses contingent on their proximity to each basin: intervals closer to isochrony exhibit positive bias, while those nearer to binary/ternary ratios exhibit negative bias through attraction toward 1:2 or 1:3. This distribution pattern suggests not an absence of regularization, but rather heterogeneity in the relative strength of competing timing mechanisms across individuals (Coissac et al. 2026). Our fixel-based results partly support this mechanistic interpretation. Isochrony biases show a positive relationship with white-matter microstructure in thalamus-precentral and striato-precentral pathways, while biases for small integer ratios show negative correlations with microstructure of the same pathways (marginally significant before correction). Secondly, this discrepancy may reflect methodological differences with iterated learning designs. Iterated and serial reproduction designs amplify weak individual biases through cumulative transmission across multiple generations: small initial biases compound over successive reproductions, producing strong population-level patterns (Griffiths et al. 2008; Kirby et al. 2008). For example, in Ravignani et al. 2016, isochrony becomes measurable after the second iteration. Our single-reproduction paradigm captures individual predispositions in their nascent form, without amplification through cultural filtering. The distribution (48.9% increasing, 51.1% decreasing isochrony) reveals heterogeneity in timing strategies that would be untangled in multi-generation designs where one attractor eventually dominates.

One main finding, which aligns with our initial hypothesis, is the positive correlation between isochrony biases and microstructure. Firstly, structural connectivity is more closely linked to the processing of sensory components (Lumaca et al. 2024), such as rhythmic categories (1:1, 2:1, 3:1), that can shape white matter through long-term acculturation (Hyde et al. 2009). This could in principle explain the absence of functional predictors for small-integer-ratio biases in the current results. Secondly, the production of isochronous interval ratios is contingent upon precise temporal coordination at the millisecond scale. Beat-based timing, the mechanism that allows us to perceive, maintain, and predict a regular pulse in music, is supported by cortico-subcortical circuits that connect basal ganglia, thalamus, and motor areas (Grahn and Rowe 2009). Prominent theoretical models, like the ASAP, propose that the basal ganglia play a significant role in beat perception by generating temporal predictions in motor regions through thalamic-motor connections (Patel and Iversen 2014). These predictions, sometimes referred to as “proto-actions,” are thought to generate signals that travel through a self-sustained loop within the motor cortico-basal-ganglia-thalamo-cortical circuit. Our results indicate that individuals with denser connections within this circuit are more likely to reproduce metronomic, isochronous rhythms. Higher fiber density may facilitate faster and more precise signal transmission within a network, reinforcing internally generated oscillations within a self-sustained loop, and imposing a metronomic temporal framework that biases rhythmic production toward isochrony (Gong et al. 2025). This pattern suggests that axonal packing density (captured by FD) plays a greater role in shaping isochrony biases than its combination with gross tract cross-section (captured by FDC). While white matter microstructure, including axon diameter and myelination, determines conduction velocity (Caminiti et al. 2013), tract cross-section additionally reflects the number of fibers available for parallel information transmission (Perge et al. 2012; Smith et al. 2022). FD, as a proxy for intra-axonal volume, is sensitive to the microstructural properties that govern conduction timing; by incorporating capacity-related variance, FDC may dilute this timing-relevant microstructural signal. Cortico motor-basal ganglia circuits are largely conserved in vertebrates (Milardi et al. 2019), and isochrony production is shared with a broad range of species, from songbirds (Roeske et al. 2020) to primates (De Gregorio et al. 2024, 2025; Eleuteri et al. 2025). These circuits may provide a stable anatomical scaffolding in rhythm perception and production, and potentially reflect the co-option of more ancient motor neurophysiology evolved for repetitive (metronomic) behaviors like locomotion (Patel and Iversen 2014). The isochrony biases they produce would have been further reinforced in our species through selection pressures for social bonding and coordination (Savage et al. 2020). The specific relationship between isochrony and white matter organization suggests that beat perception may be more deeply rooted in biology than other forms of rhythmic processes shaped by enculturation (Jacoby and McDermott 2017; Jacoby et al. 2024).

Together, these findings partly contribute to the debate about whether rhythmic biases are mainly auditory, motor, or instead arise from spontaneous interactions within auditory-motor systems (Patel and Iversen 2014). Our results point to a mechanism that involves the interplay of multiple bilaterally distributed systems. The CPM network that predicts entropy reduction included connections between networks involved in auditory timing (AUD-BG) and executive function (DAN-DMN). This suggests that rhythmic regularization depends not only on sensorimotor circuits but also on the cognitive systems that handle stimulus encoding. The structural pathways that predict isochrony (thalamo-precentral and striato-precentral tracts) connect the motor cortex with subcortical hubs that receive both auditory and cortical motor inputs. This fits with the ASAP framework (Patel and Iversen 2014; Cannon and Patel 2021), which is now supported by evidence from transcranial magnetic stimulation of the dorsal premotor cortex (Lazzari et al. 2025) and posterior parietal cortex (Ross et al. 2018). Different rhythmic biases may thus rely on different aspects of a multisystem baseline architecture that supports timing (Bedford et al. 2025).

The absence of neural predictors for small integer ratio biases (1:2, 1:3), in particular, stands in contrast to recent electrophysiological evidence demonstrating that categorization biases emerge during active listening (Barbero et al. 2025). One hypothesis is that the temporal resolution of resting-state fMRI connectivity does not achieve sufficient granularity to capture the rapid oscillatory dynamics underlying ratio categorization. Also, the emergence of ratio biases might require active engagement with rhythmic stimuli, as implemented by Barbero and colleagues (Barbero et al. 2025), whereas our brain data only capture the baseline architecture without task-evoked dynamics. The analysis in Barbero et al. was conducted at the electroencephalography (EEG) sensor level, which hinders comparison with the canonical networks analyzed here. Complementary magnetoencephalography (MEG) investigations adopting network-based perspectives would reveal how frequency-specific neural circuits attune their dynamics to the input, reorganizing their intrinsic spatiotemporal organization during stimulus processing (Rosso et al. 2025). In short, M/EEG-based network estimation in reconstructed source space may represent a promising avenue to complement our findings. Examining the balance between intrinsic and stimulus-driven dynamics may ultimately help explain the origins of intrinsic biases for small integer ratios.

More broadly, our findings may inform theoretical frameworks about the relationship between neural architecture and the emergence of musical universals through cultural transmission (Lumaca et al. 2018b). Although individual biases may appear subtle and variable in single reproduction events, they are amplified by transmission and manifest at the group level as systematic transformations of musical patterns (Ravignani et al. 2016; Lumaca and Baggio 2017; Lumaca et al. 2018a). These findings are consistent with hypotheses in cultural evolution positing that transmission involves less faithful replication and more systematic modifications guided by cognitive and neural constraints (Sperber 1996). As neural biases accumulate across generations of learners, they act as “attractors” (Claidière and Sperber 2007), gradually steering human music toward forms that reflect underlying neural predispositions (Christiansen and Müller 2015) while still allowing for considerable variation.

Complementarily to the above, one may ask whether the link we find between neurobiology and cultural transmission of rhythm is unique to humans. Just a few years ago, such a question would have been impossible to address. However, two recent developments make this line of inquiry very promising. On the one hand, animal rhythm production experiments are showing how some previously thought uniquely human capacities are in fact shared with some other species (Merchant et al. 2015; Cook et al. 2025). On the other hand, both postmortem and in vivo MRI connectivity data is being generated for nonstandard model species (Pagani et al. 2023; Flem et al. 2025; Cook et al. 2026). In brief, in a few years, it may be possible to do rhythm production tasks with other species while linking the resulting analogous or homologous brain networks to those we find here.

## Limitations

This work presents two main limitations. First, the proposed link between neural variability and broader cultural phenomena should be regarded as exploratory at this stage. Our inference rests on limited evidence, as the participant sample was largely restricted to individuals from WEIRD (Western, Educated, Industrialized, Rich, and Democratic) societies. Although the core perceptual and neural mechanisms we identify are hypothesized to be universal (Drake 1998; Drake and Bertrand 2001), a large-scale, multisite neuroimaging study is essential to determine the extent to which the observed brain–behavior relationship is culturally dependent or reflects a fundamental universal.

Second, the use of static functional connectivity, while appropriate for linking the origins of rhythmic biases to intrinsic network connectivity, precludes any mechanistic account of their temporal unfolding. This approach was deliberate: we primarily aimed to test whether these biases are partly rooted in the human baseline architecture. Future research employing task-based M/EEG with high temporal resolution will be crucial to identify their underlying neural dynamics, such as the dominant oscillation frequencies (eg beta band) and cross-regional phase-synchronization patterns that bias rhythmic behavior toward regularization.

## Conclusion

The present study provides a neurobiological account for rhythmic regularization biases observed in humans. By demonstrating a statistical association between intrinsic brain architecture and rhythm processing and transmission, it reveals some of the neurobiological boundaries within which cultural variation can occur in music. All humans share cortico-striatal circuits that favor predictable rhythms of small integer ratios, but the relative strength of such biases is expected to vary across individuals due to various factors, including genetic polymorphisms, developmental trajectories, and experience-dependent plasticity. Regardless of which of these sources is predominant, they all shape individual biases in rhythm processing. When such biases are unevenly distributed across populations, they may channel symbolic systems down different cultural paths, albeit within a constrained space defined by the fundamental architecture of the human brain—a framework previously proposed to explain cross-cultural variation in symbolic systems (Dediu and Ladd 2007). Our cross-sectional design and reliance on a WEIRD sample preclude definitive claims about the origins of this variability or about the generalizability of these brain–behavior relationships across human groups. However, the general architecture of cortico-striatal circuits is highly conserved (Haber 2016; Heilbronner et al. 2016): if it shapes rhythmic biases, as our data suggest, we would expect such constraints to operate universally regardless of cultural background. Within this paradigm, music universals may reflect enduring influences of human neurobiology, shaped by generations of music learners and producers through oral transmission (Lumaca et al. 2018b).

## Supplementary Material

CerCor_2026-00007_SupplMat_R1_bhag091

## Data Availability

Scripts, code, and behavioral data used for all analyses are available from https://github.com/Jellevanderwerff/rhythmic_universals_in_brain_network_organization. Neuroimaging data is available upon request from the corresponding author.
