# Supplementary material for: Cortico-striatal network architecture shapes rhythmic biases in music
Source: Cereb Cortex. 2026 Oct 6;36(10):bhag091. doi: 10.1093/cercor/bhag091 (PMC13641083; doi:10.1093/cercor/bhag091)
Supplement: Supplementary file 1 [file cercor_2026-00007_supplmat_r1_bhag091.docx]

**Supplementary Material**

**Cortico-striatal network architecture shapes rhythmic biases in music**

Massimo Lumaca ^*,1^, Jelle van der Werff ^2^, Peter E. Keller ^1,3^, Sonja A. Kotz ^4,5^, Giosuè Baggio ^6^, Mattia Rosso ^1,7^, Marcelo Kakihara ^8^, Mathilde Martine ^9^, Peter Vuust ^#,1^, Andrea Ravignani ^#,1,2,10,11,12,*^

1. Center for Music in the Brain, Aarhus University & The Royal Academy of Music Aarhus/Aalborg, Aarhus, Denmark

2. Department of Human Neurosciences, Sapienza University of Rome, Rome, Italy

3. The MARCS Institute for Brain, Behaviour and Development, Western Sydney University, Sydney, Australia

4. Faculty of Psychology and Neuroscience, Dept. of Neuropsychology & Psychopharmacology, Maastricht University

5. Department of Neuropsychology, Max-Planck-Institute for Human Cognitive and Brain Sciences, Leipzig, Germany

6. Language Acquisition and Language Processing Lab, Norwegian University of Science and Technology, Trondheim, Norway

7. IPEM Institute for Systematic Musicology, Ghent University, Ghent, Belgium

8. Kyoto University, Kyoto, Japan

9. ESPCI Paris, Paris, France

10. Research Center of Neuroscience ‘‘CRiN-Daniel Bovet’’, Sapienza University of Rome, Rome, Italy

11. Institute of Cognitive Sciences and Technologies, National Research Council, Rome, Italy

12. Comparative Bioacoustics Group, Max Planck Institute for Psycholinguistics, Nijmegen, The Netherlands

* corresponding authors

\# co-senior authors

Mailing address: Massimo Lumaca: Dept. of Clinical Medicine, Health, Aarhus University Universitetsbyen 3 / building 1710 8000 Aarhus C

Email: massimo.lumaca@clin.au.dk

**Supplementary Figures**

**Fig. S1.** Pie charts showing the distribution of musical training duration and number of instruments played among participants across the Gold-MSI musical training subscales (N=47). Percentages in the legend indicate the proportion of participants categorized by the relative subscale. The majority of our participants are classified as non-musicians (less than 6 years of musical training) (Zhang et al. 2018).

**Figure S2.** Edit (Levenshtein) distance between stimulus and response sequences by condition. The most challenging condition was slow tempo with 5 events. A one-way ANOVA across trials and participants shows that condition significantly influences edit distance (*F*(3) = 244, p< .001, 95% CI: [0.08, 1.00], η^2^= 0.09). In the plot, each dot represents a participant, showing their mean edit distance averaged across all trials within a given condition.

**Figure S3.** Correlation matrix with regression-line scatterplots between isochrony, binary/ternary ratios, and entropy reduction. Each point is one participant (N=47). One asterisk indicates *p* < .05, two asterisks indicate *p* < .01, and three indicate *p* < .001.

**Figure S4. Functional anatomy of the CPM model (k=5) predicting Entropy difference. A)** Functional connectivity patterns of the negative model network predicting response-minus-stimulus changes in rhythmic entropy using a robust 5-fold cross-validation. Top: Glass brain plots (right and top views) with nodes (spheres) sized by degree (number of edges). Bottom: Circular connectogram representing the distribution of links between brain regions. Nodes (spheres) are color-coded by network affiliation: Visual (VIS, deep purple), Motor (MOT, steel blue), Dorsal Attention (DAN, dark green), Ventral Attention (VAN, orange), Limbic (LIM, light green), Auditory (AUD, magenta), Default Mode (DMN, red), Basal Ganglia-Thalamic (BG-TH, gray), sub-limbic (SUB-LIM, soft green), cerebellar (CB, warm orange). **B)** Correlation between actual entropy difference scores and those predicted by CPM using negative features (N = 47). Gray shading: 95% CI. Model validation: r= 0.38, p_perm= .01. **C)** Between-network connections count matrix. Cells indicate the number of connections between network pairs; lighter colors represent a larger number of connections. **D)** Matrices with p-values obtained from nonparametric permutation statistics shuffling node-to-network mappings. On the left, raw p-values. On the right, p-values are corrected with False Discovery Rate.

**Figure S5. Comparison of between-network FC in participant groups split by median entropy difference. A)** Participants were divided into two groups based on median split of entropy change scores for visualization purposes only: 'high regularizers' (entropy change < median; n = 24) showed greater entropy reduction, while 'low regularizers' (entropy change ≥ median; n = 23) showed lesser reduction. This division was not used in statistical analyses, which treated entropy change as a continuous variable. Numbers reflect mean inter-network functional connectivity (FC); yellow colors represent stronger, positive FC; dark blue colors represent stronger, negative FC (i.e., network anti-coupling).

**Figure S6. A)** Functional connectivity patterns of the negative model network predicting response-minus-stimulus changes in rhythmic entropy using a robust 10-fold cross-validation. Top: Glass brain plots (right and top views) with nodes (spheres) sized by degree (number of edges). Bottom: Circular connectogram representing the distribution of links between brain regions. Nodes (spheres) are color-coded by network affiliation: Visual (VIS, deep purple), Motor (MOT, steel blue), Dorsal Attention (DAN, dark green), Ventral Attention (VAN, orange), Limbic (LIM, light green), Auditory (AUD, magenta), Default Mode (DMN, red), Basal Ganglia-Thalamic (BG-TH, gray), sub-limbic (SUB-LIM, soft green), cerebellar (CB, warm orange). **B)** Correlation between actual entropy difference scores and those predicted by CPM using negative features (N = 47). Gray shading: 95% CI. Model validation: r= 0.36, p_perm= .03. **C)** Between-network connections count matrix. Cells indicate the number of connections between network pairs; lighter colors represent a larger number of connections. **D)** Matrices with p-values obtained from nonparametric permutation statistics shuffling node-to-network mappings. On the left, raw p-values. On the right, p-values are corrected with False Discovery Rate.

**Figure S7. The five white-matter tracts selected for fixel-based analyses and fiber-based metrics.** The selected white matter tracts include inferior, middle, and superior cerebellar peduncle (CP); striato-precentral (ST-PREC), striato-premotor (ST-PREM), thalamo-premotor (T-PREM), thalamo-precentral (T-PREC). These white-matter tracts belong to two interfacing systems: the striatal-thalamic-motor cortical network and the cerebellar network, both of which are involved in rhythmic perception and production (Penhune et al. 1998; Grahn and Rowe 2009; Kotz et al. 2018). For these bilateral tracts, we computed the average for three fixel-based metrics: fiber density (fd; degree of axonal packing in a fiber population); fiber cross-section (fc; cross-sectional size of a fiber population); fiber density and cross-section (fdc; quantifying the total intra-axonal volume of a fiber bundle).

**Supplementary Tables**

**Table S1.** Descriptive statistics for Musical Ear Test (MET), Goldsmiths Musical Sophistication Index (Gold-MSI), and Wechsler Adult Intelligence Scale IV edition (WAIS-IV) (N=47).

|  | *Mean* | *Std* | *Score Range (theoretical max)* |
| --- | --- | --- | --- |
| **MET** |  |  |  |
| Rhythmic | 37.68 | 5.17 | 25 - 48 (52) |
| **Gold-MSI** |  |  |  |
| Musical training | 2 | 1.14 | 1-5.71 (49) |
| **WAIS-IV** |  |  |  |
| Working memory | 99.75 | 14.50 | 63-132 |

**Table S2.** Descriptive statistics by condition for all participants (N=47). The experiment employed a 2×2 within-subjects design: Tempo (400 ms vs. 600 ms mean inter-onset intervals) and Length (4 vs. 5 sounds). This resulted in four conditions: 4004 (400 ms mean IOI and 4 sounds); 4005 (400 ms and 5 sounds); 6004 (600 ms and 4 sounds); 6005 (600 ms and 5 sounds).

| **Entropy** | *Condition* | *Mean* | *Standard deviation* | *Median* |
| --- | --- | --- | --- | --- |
|  | 4004 | -0.075 | 0.073 | -0.070 |
|  | 4005 | -0.083 | 0.073 | -0.095 |
|  | 6004 | -0.080 | 0.120 | -0.093 |
|  | 6005 | -0.064 | 0.103 | -0.061 |
|  | | | | |
| **Isochrony** | *Condition* | *Mean* | *Standard deviation* | *Median* |
|  | 4004 | 0.344 | 1.393 | 0.382 |
|  | 4005 | 0.164 | 1.092 | 0.272 |
|  | 6004 | -0.023 | 2.016 | 0.209 |
|  | 6005 | -0.249 | 1.885 | -0.521 |
|  | | | | |
| **Binary or ternary ratios** | *Condition* | *Mean* | *Standard deviation* | *Median* |
|  | 4004 | 1.201 | 1.397 | 0.943 |
|  | 4005 | 1.185 | 1.429 | 0.921 |
|  | 6004 | 1.236 | 1.726 | 1.039 |
|  | 6005 | 1.388 | 1.568 | 1.247 |

**Table S3.** Repeated-measures ANOVA with Greenhouse-Geisser correction to test the effect of stimulus tempo and length on behavioural measures. The experiment employed a 2×2 within-subjects design: Tempo (400 ms vs. 600 ms mean inter-onset intervals) and Length (4 vs. 5 sounds). F values, degrees of freedom (dof), and p-values are reported for main effects (tempo and length) and interactions (tempo x length).

| **Entropy** | *F-value* | *dof* | *p-value* |
| --- | --- | --- | --- |
| Tempo | 0.4272 | 46 | 0.51 |
| Length | 0.2068 | 46 | 0.65 |
| Tempo x Length | 2.4422 | 46 | 0.12 |
|  | | | |
| **Isochrony** | *F-value* | *dof* | *p-value* |
| Tempo | 3.5849 | 46 | 0.06 |
| Length | 1.4441 | 46 | 0.23 |
| Tempo x Length | 0.0305 | 46 | 0.86 |
|  | | | |
| **Binary or ternary ratios** | *F-value* | *dof* | *p-value* |
| Tempo | 0.3520 | 46 | 0.55 |
| Length | 0.1959 | 46 | 0.66 |
| Tempo x Length | 0.2937 | 46 | 0.59 |

**Table S4.** List and abbreviations of 84 Desikan atlas’s parcels and their assignment to ten resting-state networks: seven cortical (visual, VIS; limbic, LIM; default mode network, DMN; dorsal attention network, DAN; ventral attention network, VAN; motor network, MOT; auditory network, AUD) [(Shirer et al. 2012)](https://paperpile.com/c/mNbqvW/XF7PT) and three subcortical (basal ganglia, BG; limbic subcortical, LSC; cerebellar, CB).

| **Region’s name (DK atlas)** | **Network** |
| --- | --- |
| lh Banks superior temporal sulcus | AUD |
| lh Caudal anterior-cingulate cortex | DAN |
| lh Caudal middle frontal gyrus | VAN |
| lh Cuneus cortex | VIS |
| lh Entorhinal cortex | LIM |
| lh Fusiform gyrus | VIS |
| lh Inferior parietal cortex | DMN |
| lh Inferior temporal gyrus | DAN |
| lh Isthmus cingulate cortex | DMN |
| lh Lateral occipital cortex | VIS |
| lh Lateral orbital frontal cortex | DMN |
| lh Lingual gyrus | VIS |
| lh Medial orbital frontal cortex | DMN |
| lh Middle temporal gyrus | DMN |
| lh Parahippocampal gyrus | DMN |
| lh Paracentral lobule | MOT |
| lh Pars opercularis | DAN |
| lh Pars orbitalis | DAN |
| lh Pars triangularis | DAN |
| lh Pericalcarine cortex | VIS |
| lh Postcentral gyrus | MOT |
| lh Posterior-cingulate cortex | DMN |
| lh Precentral gyrus | MOT |
| lh Precuneus cortex | DMN |
| lh Rostral anterior cingulate cortex | DMN |
| lh Rostral middle frontal gyrus | VAN |
| lh Superior frontal gyrus | DMN |
| lh Superior parietal cortex | DAN |
| lh Superior temporal gyrus | AUD |
| lh Supramarginal gyrus | VAN |
| lh Frontal pole | LIM |
| lh Temporal pole | LIM |
| lh Transverse temporal cortex | AUD |
| lh Insula | VAN |
| lh Cerebellum | CB |
| lh Thalamus | BG |
| lh Caudate | BG |
| lh Putamen | BG |
| lh Pallidum | BG |
| lh Hippocampus | LSC |
| lh Amygdala | LSC |
| lh Accumbens | LSC |
| rh Thalamus | BG |
| rh Caudate | BG |
| rh Putamen | BG |
| rh Pallidum | BG |
| rh Hippocampus | LSC |
| rh Amygdala | LSC |
| rh Accumbens | LSC |
| rh Banks superior temporal sulcus | AUD |
| rh Caudal anterior-cingulate cortex | DAN |
| rh Caudal middle frontal gyrus | VAN |
| rh Cuneus cortex | VIS |
| rh Entorhinal cortex | LIM |
| rh Fusiform gyrus | VIS |
| rh Inferior parietal cortex | DMN |
| rh Inferior temporal gyrus | DAN |
| rh Isthmus cingulate cortex | DMN |
| rh Lateral occipital cortex | VIS |
| rh Lateral orbital frontal cortex | DMN |
| rh Lingual gyrus | VIS |
| rh Medial orbital frontal cortex | DMN |
| rh Middle temporal gyrus | DMN |
| rh Parahippocampal gyrus | DMN |
| rh Paracentral lobule | MOT |
| rh Pars opercularis | DAN |
| rh Pars orbitalis | DAN |
| rh Pars triangularis | DAN |
| rh Pericalcarine cortex | VIS |
| rh Postcentral gyrus | MOT |
| rh Posterior-cingulate cortex | DMN |
| rh Precentral gyrus | MOT |
| rh Precuneus cortex | DMN |
| rh Rostral anterior cingulate cortex | DMN |
| rh Rostral middle frontal gyrus | VAN |
| rh Superior frontal gyrus | DMN |
| rh Superior parietal cortex | DAN |
| rh Superior temporal gyrus | AUD |
| rh Supramarginal gyrus | VAN |
| rh Frontal pole | LIM |
| rh Temporal pole | LIM |
| rh Transverse temporal cortex | AUD |
| rh Insula | VAN |
| rh Cerebellum | CB |

**Table S5.** Functional connection identified with connectome-based predictive modeling (CPM) for entropy difference using 5-fold cross-validation. Network-to-network connections (N=27) are arranged by number in descending order.

| **Network pair** | **Region 1** | **Region 2** |
| --- | --- | --- |
| *Between-network connections* | | |
| DAN - DMN | lh Pars Orbitalis | rh Inferior Parietal cortex |
|  |  | rh Medial Orbital Frontal cortex |
|  |  | rh Precuneus |
|  |  | rh Rostral Anterior Cingulate cortex |
|  | lh Pars Triangularis | lh Rostral Anterior Cingulate cortex |
|  |  | rh Rostral Anterior Cingulate cortex |
|  |  | rh Superior Frontal cortex |
|  | lh Pars Opercularis | rh Rostral Anterior Cingulate cortex |
|  | rh Pars Orbitalis | rh Medial Orbital Frontal cortex |
|  | rh Inferior Temporal | rh Inferior Parietal cortex |
| AUD-BG | lh Banks superior temporal sulcus | lh Thalamus |
|  | lh Banks superior temporal sulcus | rh Putamen |
|  | lh Superior temporal gyrus | rh Putamen |
|  | rh Superior temporal gyrus | rh Thalamus |
| VIS-AUD | lh Fusiform gyrus | lh Banks superior temporal sulcus |
|  |  | rh Banks superior temporal sulcus |
| VAN-DMN | lh Caudal Middle Frontal gyrus | lh Inferior Parietal cortex |
|  |  | rh Inferior Parietal cortex |
| MOT-BG | lh Postcentral gyrus | rh Putamen |
| MOT-LBS | lh Precentral gyrus | rh Accumbens |
| DAN-AUD | lh Inferior Temporal gyrus | rh Banks superior temporal sulcus |
| *Within-network connections* | | |
| DMN | lh Superior Frontal gyrus | rh Precuneus |
|  |  | rh Rostral Anterior Cingulate cortex |
|  |  | lh Rostral Anterior Cingulate cortex |
| DAN | lh Pars Triangularis | rh Caudal Anterior Cingulate |
|  |  | rh Pars Triangularis |
| BG | lh Pallidum | rh Putamen |

**Table S6.** Results from nonparametric permutations in entropy reduction using 5-fold cross-validation in CPM. Only statistics for network pairs with actual connections are reported.

| Network pair | Connection count | Z score | Confidence intervals  (lower, upper) | pFDR adjusted |
| --- | --- | --- | --- | --- |
| DAN - DMN | 10 | 5.55 | 0,5 | < 0.001 |
| AUD-BG | 4 | 5.35 | 0,2 | o.01 |
| VIS-AUD | 2 | 2.16 | 0,2 | 0.19 |
| VAN-DMN | 2 | 0.62 | 0,4 | 0.40 |
| MOT-BG | 1 | 0.99 | 0,2 | 0.40 |
| MOT-LBS | 1 | 1.22 | 0,2 | 0.40 |
| DAN-AUD | 1 | 0.50 | 0,3 | 0.42 |

**Table S7.** Functional connection identified with connectome-based predictive modeling (CPM) for entropy difference using 10-fold cross-validation. Network-to-network connections (N=45) are arranged by number in descending order.

| **Network pair** | **Region 1** | **Region 2** |
| --- | --- | --- |
| *Between-network connections* | | |
| DAN - DMN | lh Pars Orbitalis | lh Precuneus |
|  |  | lh Inferior Parietal cortex |
|  |  | lh Isthmus Cingulate |
|  |  | lh Lateral Orbitofrontal cortex |
|  |  | lh Medial Orbitofrontal cortex |
|  |  | rh Precuneus |
|  |  | rh Rostral Anterior Cingulate cortex |
|  | lh Pars Triangularis | lh Rostral Anterior Cingulate cortex |
|  |  | rh Rostral Anterior Cingulate cortex |
|  |  | lh Superior Frontal cortex |
|  | rh Pars Orbitalis | rh Medial Orbitofrontal cortex |
|  | lh Pars Opercularis | rh Rostral Anterior Cingulate cortex |
|  | lh Inferior Parietal cortex | rh Inferior Parietal cortex |
| AUD-BG | lh Banks superior temporal sulcus | lh Thalamus |
|  |  | rh Thalamus |
|  |  | rh Putamen |
|  | lh Superior Temporal gyrus | rh Thalamus |
|  |  | rh Putamen |
|  | rh Superior Temporal gyrus | rh Thalamus |
| BG-MOT | rh Putamen | lh Postcentral |
|  |  | rh Paracentral |
|  |  | rh Postcentral |
| VIS-AUD | lh Fusiform gyrus | lh Banks superior temporal sulcus |
|  |  | rh Banks superior temporal sulcus |
| VAN-DMN | lh Caudal Middle Frontal gyrus | lh Inferior Parietal cortex |
|  |  | rh Inferior Parietal cortex |
| *Within-network connections* | | |
| DMN | lh Superior Frontal | lh Precuneus |
|  |  | lh Rostral Anterior Cingulate cortex |
|  |  | rh Precuneus |
|  |  | rh Rostral Anterior Cingulate cortex |
|  | lh Middle Temporal | rh Lateral Orbitofrontal |
| BG | lh Thalamus | rh Pallidum |
|  | lh Pallidum | rh Putamen |

**Table S8.** Results from nonparametric permutations in entropy reduction using 10-fold cross-validation in CPM. Only statistics for network pairs with actual connections are reported.

| Network pair | Connection count | Z score | Confidence intervals  (lower, upper) | P-value (raw) | P-value (adjusted) |
| --- | --- | --- | --- | --- | --- |
| DMN-DAN | 13 | 4.98 | 0,8 | <.001 | <.001 |
| AUD-BG | 6 | 6.99 | 0,2 | <.001 | <.001 |
| MOT-BG | 3 | 2.85 | 0,3 | 0.03 | 0.14 |
| VIS-AUD | 2 | 1.27 | 0,3 | 0.19 | 0.55 |
| DMN-VAN | 2 | -0.01 | 0,5 | 0.59 | 0.68 |
| CB-VAN | 1 | 1.72 | 0,1 | 0.19 | 0.55 |
| AUD-MOT | 1 | 0.80 | 0,2 | 0.35 | 0.64 |
| LBS-MOT | 1 | 0.81 | 0,2 | 0.33 | 0.64 |
| LBS-LIM | 1 | 0.71 | 0,3 | 0.36 | 0.64 |
| BG-LIM | 1 | 0.49 | 0,3 | 0.42 | 0.65 |
| VAN-VIS | 1 | -0.08 | 0,4 | 0.63 | 0.68 |
| AUD-DAN | 1 | 0.06 | 0,3 | 0.59 | 0.68 |
| LBS-DAN | 1 | 0.04 | 0,4 | 0.57 | 0.68 |
| VAN-DAN | 1 | -0.20 | 0,4 | 0.69 | 0.69 |

**Table S9.** Multiple regression statistics to examine the relationship between behavioural measures and fiber density (FD) for the five white matter tracts of interest. Raw p-values (before FDR correction) are reported. * pFDR ≤ 0.05.

| Behavioral measure | White matter tract | P-value | Coefficient | Standard Error | T-Statistic | Confidence intervals  (lower, upper) | F-Statistic | Adjusted R-Squared |
| --- | --- | --- | --- | --- | --- | --- | --- | --- |
| Entropy differences | T_PREM | 0.317 | -0.676 | 0.667 | -1.013 | [-2.021, 0.670] | 0.58 | -0.019 |
|  | T_PREC | 0.016 | -1.87 | 0.742 | -2.521 | [-3.366, -0.373] | 3.254 | 0.093 |
|  | ST_PREM | 0.232 | -0.842 | 0.694 | -1.212 | [-2.243, 0.559] | 0.803 | -0.009 |
|  | ST_PREC | 0.018 | -2.097 | 0.853 | -2.457 | [-3.819, -0.374] | 3.093 | 0.087 |
|  | CP | 0.564 | -0.597 | 1.025 | -0.582 | [-2.664, 1.471] | 0.235 | -0.036 |
| Binary/ternary ratios introduced | T_PREM | 0.12 | -0.694 | 0.437 | -1.588 | [-1.576, 0.188] | 1.877 | 0.038 |
|  | T_PREC | 0.044 | -1.049 | 0.505 | -2.075 | [-2.068, -0.0287] | 2.794 | 0.075 |
|  | ST_PREM | 0.25 | -0.54 | 0.464 | -1.165 | [-1.476, 0.395] | 1.28 | 0.013 |
|  | ST_PREC | 0.086 | -1.033 | 0.587 | -1.759 | [-2.218, 0.152] | 2.172 | 0.051 |
|  | CP | 0.082 | -1.18 | 0.662 | -1.784 | [-2.515, 0.1548] | 2.217 | 0.052 |
| Isochrony introduced | T_PREM | 0.235 | 0.694 | 0.577 | 1.204 | [-0.469, 1.857] | 0.825 | -0.008 |
|  | T_PREC | 0.002* | 2.054 | 0.614 | 3.343 | [0.814, 3.294] | 5.71 | 0.176 |
|  | ST_PREM | 0.237 | 0.723 | 0.604 | 1.198 | [-0.4945, 1.941] | 0.818 | -0.008 |
|  | ST_PREC | 0.003* | 2.233 | 0.714 | 3.126 | [0.791, 3.674] | 5.006 | 0.154 |
|  | CP | 0.2 | 1.141 | 0.876 | 1.302 | [-0.627, 2.909] | 0.948 | -0.002 |

**Table S10.** Multiple regression statistics to examine the relationship between behavioural measures and fiber cross-section (FC) for the five white matter tracts of interest. Raw p-values (before FDR correction) are reported. * pFDR ≤ 0.05.

| Behavioral Variable | Base Metric | P-value | Coefficient | Standard Error | T-Statistic | Confidence intervals  (lower, upper) | F-Statistic | Adjusted R-Squared |
| --- | --- | --- | --- | --- | --- | --- | --- | --- |
| Entropy differences | T_PREM | 0.591 | 0.182 | 0.335 | 0.542 | [-0.495, 0.858] | 0.38 | -0.044 |
|  | T_PREC | 0.467 | 0.18 | 0.245 | 0.734 | [-0.315, 0.675] | 0.463 | -0.038 |
|  | ST_PREM | 0.773 | 0.087 | 0.299 | 0.291 | [-0.517, 0.691] | 0.309 | -0.049 |
|  | ST_PREC | 0.548 | 0.153 | 0.254 | 0.605 | [-0.358, 0.665] | 0.404 | -0.042 |
|  | CP | 0.076 | 0.417 | 0.229 | 1.823 | [-0.044, 0.879] | 1.41 | 0.027 |
| Binary/ternary ratios introduced | T_PREM | 0.668 | -0.096 | 0.223 | -0.432 | [-0.546, 0.353] | 0.808 | -0.013 |
|  | T_PREC | 0.93 | -0.014 | 0.164 | -0.088 | [-0.345, 0.3165] | 0.745 | -0.018 |
|  | ST_PREM | 0.862 | -0.035 | 0.199 | -0.176 | [-0.436, 0.366] | 0.753 | -0.017 |
|  | ST_PREC | 0.968 | -0.007 | 0.169 | -0.04 | [-0.348, 0.334] | 0.743 | -0.018 |
|  | CP | 0.394 | -0.135 | 0.157 | -0.861 | [-0.451, 0.1814] | 1.003 | 0 |
| Isochrony introduced | T_PREM | 0.981 | 0.007 | 0.294 | 0.023 | [-0.586, 0.600] | 0.114 | -0.064 |
|  | T_PREC | 0.546 | -0.131 | 0.215 | -0.609 | [-0.564, 0.303] | 0.239 | -0.055 |
|  | ST_PREM | 0.721 | 0.094 | 0.262 | 0.36 | [-0.434, 0.622] | 0.158 | -0.061 |
|  | ST_PREC | 0.595 | -0.119 | 0.222 | -0.536 | [-0.567, 0.329] | 0.211 | -0.057 |
|  | CP | 0.175 | -0.281 | 0.203 | -1.38 | [-0.691, 0.130] | 0.754 | -0.017 |

**Table S11.** Multiple regression statistics to examine the relationship between behavioural measures and fiber density and cross-section (FBC) for the five white matter tracts of interest. Raw p-values (before FDR correction) are reported. * pFDR ≤ 0.05.

| Behavioral Variable | Base Metric | P-value | Coefficient | Standard Error | T-Statistic | Confidence intervals  (lower, upper) | F-Statistic | Adjusted R-Squared |
| --- | --- | --- | --- | --- | --- | --- | --- | --- |
| Entropy differences | T_PREM | 0.732 | -0.162 | 0.47 | -0.344 | [-1.110, 0.787] | 0.32 | -0.049 |
|  | T_PREC | 0.334 | -0.403 | 0.412 | -0.978 | [-1.23, 0.429] | 0.605 | -0.028 |
|  | ST_PREM | 0.626 | -0.22 | 0.448 | -0.49 | [-1.124, 0.685] | 0.362 | -0.046 |
|  | ST_PREC | 0.39 | -0.392 | 0.452 | -0.868 | [-1.305, 0.520] | 0.536 | -0.033 |
|  | CP | 0.479 | 0.329 | 0.461 | 0.715 | [-0.601, 1.259] | 0.454 | -0.039 |
| Binary/ternary ratios introduced | T_PREM | 0.151 | -0.446 | 0.305 | -1.464 | [-1.061, 0.169] | 1.495 | 0.033 |
|  | T_PREC | 0.205 | -0.35 | 0.272 | -1.288 | [-0.898, 0.1986] | 1.325 | 0.022 |
|  | ST_PREM | 0.302 | -0.308 | 0.295 | -1.044 | [-0.902, 0.287] | 1.125 | 0.008 |
|  | ST_PREC | 0.256 | -0.343 | 0.298 | -1.152 | [-0.945, 0.258] | 1.208 | 0.014 |
|  | CP | 0.108 | -0.49 | 0.298 | -1.644 | [-1.092, 0.1118] | 1.692 | 0.045 |
| Isochrony introduced | T_PREM | 0.475 | 0.295 | 0.409 | 0.721 | [-0.531, 1.1209] | 0.289 | -0.051 |
|  | T_PREC | 0.188 | 0.478 | 0.357 | 1.339 | [-0.243, 1.198] | 0.717 | -0.02 |
|  | ST_PREM | 0.411 | 0.324 | 0.39 | 0.83 | [-0.463, 1.110] | 0.346 | -0.047 |
|  | ST_PREC | 0.253 | 0.455 | 0.393 | 1.16 | [-0.337, 1.2485] | 0.566 | -0.03 |
|  | CP | 0.943 | -0.029 | 0.405 | -0.072 | [-0.848, 0.789] | 0.116 | -0.064 |
